# One Pot and Metal-Free Approach to 3-(2-Hydroxybenzoyl)-1-aza-anthraquinones

**DOI:** 10.3390/molecules24163017

**Published:** 2019-08-20

**Authors:** Jiaqi Yuan, Qian He, Shanshan Song, Xiaofei Zhang, Zehong Miao, Chunhao Yang

**Affiliations:** 1 State Key Laboratory of Drug Research, Shanghai Institute of Materia Medica, Chinese Academy of Sciences, 555 Zuchongzhi Road, Shanghai 201203, China; 2 School of Pharmacy, University of Chinese Academy of Sciences, No. 19A Yuquan Road, Beijing 100049, China

**Keywords:** 3-(2-hydroxybenzoyl)-1-aza-anthraquinones, chromones, anti-proliferation agent

## Abstract

Herein, a direct strategy to synthesize 3-(2-hydroxybenzoyl)-1-aza-anthraquinones with excellent efficiency, mild conditions, and benign functional group compatibility was reported. A variety of 3-formylchromone compounds were employed as compatible substrates and this protocol gave the 3-(2-hydroxybenzoyl)-1-aza-anthraquinone derivatives in good to excellent yields without inert gas and expensive transition metal catalysts. Some compounds displayed good anti-proliferative activities.

## 1. Introduction

**Figure 1:**
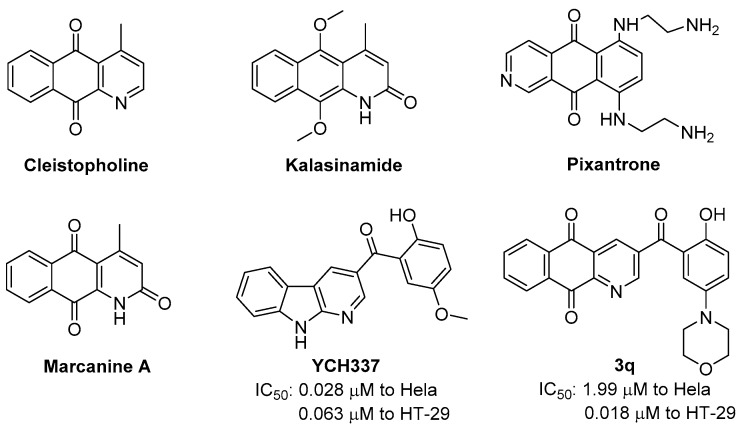
Biologically active aza-anthraquinone derivatives and YCH337.

Anthraquinone, as a classical pharmacophore, has been applied in drug discovery for many years, [1,2,3] and this scaffold contributes to a great number of important drugs, such as Mitoxantrone [4], Doxorubicin [5], and Daunorubicin [6]. Aza-anthraquinones are crucial analogues of anthraquinones, which have attracted considerable attention for their pronounced biological activities (Figure 1) [7,8,9]. For example, Marcanine A was first isolated from the stem bark of *Goniothalamus marcanii* in 1999 and was proved to have significant cytotoxicity against human tumor cell lines [10,11]. Kalasinamide was discovered in *Polyalthia suberosa* in 2000 and turned out to be a mild anti-HIV agent. [8,12]

**Figure 2:**
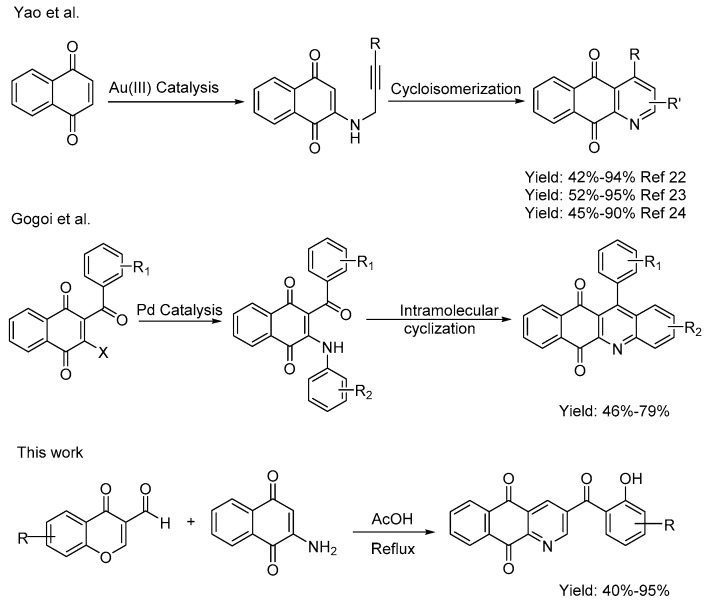
Different strategies to synthesize 1-aza-anthraquinone derivatives.

As far as we know, there are some synthetic routes that have been elaborated to construct 1-aza-anthraquinones, such as Diels–Alder reactions [13,14,15,16], Friedel–Crafts reactions [17,18], Michael-type addition followed by ring expansion [19,20], and 6-*exo-trig* radical cyclization of *N*-(2-alkenylaryl)-substituted enamines [21]. Despite the great potential of these methods, the use of toxic metal agents, inconvenient availability of starting materials, as well as poor regioselectivity and low yields of these reactions limited their wide applications. Recently, Yao et al. developed a range of methods to assemble 1-aza-anthraquinone skeleton from N-propargylamino quinone through a 6-*endo*-*dig* electrophilic cyclization (Figure 2) [22,23,24]. Gogoi et al. reported a sequential Pd-catalyzed C-N bond formation, followed by intramolecular cyclization to synthesize 1-aza-anthraquinones [25]. These methods are efficient strategies to assemble 1-aza-anthraquinone scaffold with regioselectivity and good yields. Meanwhile, the preparation of N-propargylamino quinone and 2-Benzoyl-3-halonaphthalene-1,4-diones, which were catalyzed by expensive transition metal, limited the application of these methods in a sense [24,25]. Therefore, a new practical and convenient approach for 1-aza-anthraquinone is still highly desired.

Our lab has been focusing on the construction of nitrogen-containing heterocyclic frameworks derived from chromones for several years [26,27,28,29,30,31]. Previously, one synthetic compound YCH337 prepared from chromone was found to be a potent anti-proliferation agent against tumor cells with an averaged IC_50_ of 0.3 µM by targeting both microtubule and Topoisomerase 2 (Figure 1) [32]. Inspired by our previous works and great enthusiasm on the converged structure of YCH337 and potential bioactive 1-aza-anthraquinone scaffold, we designed and synthesized novel tricyclic 3-(2-hydroxybenzoyl)-1-aza-anthraquinone derivatives as YCH337 analogues.

## 2. Results and Discussion

3-Formylchromones are representative building blocks, which are usually employed to prepare varieties of heterocyclic systems with enamine-type compounds [33,34,35,36,37,38]. Accordingly, we chose 3-formylchromone (**1a**) and 2-amino-naphthalene-1,4-dione (**2a**) as the template substrates to optimize the reaction conditions (Table 1). Surprisingly, the initial attempt gave the targeting product **3a** with 35% yield after stirring in acetic acid for 24 h at 80 °C (Table 1, entry 1). Based on this result, different reaction temperatures were examined (Table 1, entries 1 to 3). By the increasing reaction temperature, the yield was remarkably improved and the reaction time was shortened to 4 h under reflux. Then, a variety of solvents were screened for the reaction and the results showed that acetic acid was superior to other solvents (Table 1, entries 4 to 8). In addition, further investigations were conducted on the ratio of reactants, finding that 1.2 eq of 3-formylchromone gave a better yield (Table 1, entry 3, entries 9 to 12). Therefore, after the screening of the temperature, solvent, and reagents’ ratio, the optimized reaction conditions were established (Table 1, entry 10).

**Figure 3:**
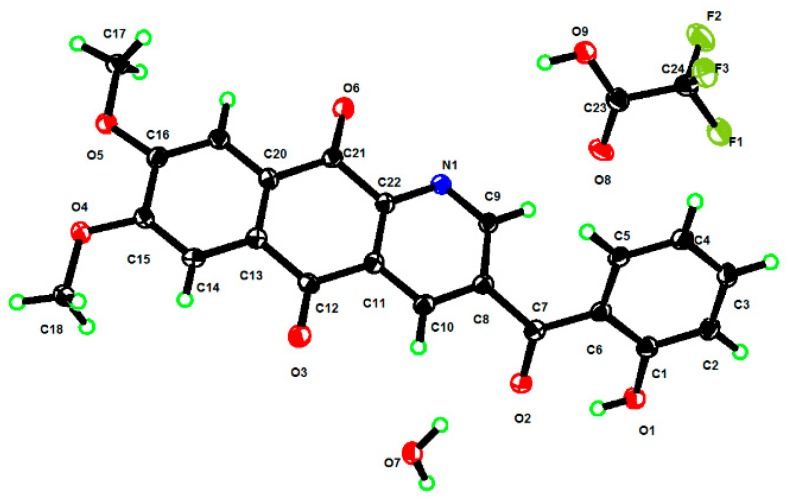
The single crystal structure of **3t**.

With the optimized reaction conditions in hand, we next explored the substrates’ scope and the generality of the reaction with different 3-formylchromones. To our delight, this protocol was applicable to a series of 3-formylchromones under the optimized reaction conditions, providing the corresponding products **3a**–**3t** with moderate to excellent yields (Table 2, 40–95%). All substituted 3-formylchromones with electron-donating groups (methyl, methoxyl groups) or electron-withdrawing groups (chloro, bromo, fluoro, cyano, and nitro groups) could be transformed into the desired compounds (**3a**–**3q**, 40–90%). When there was an electron-donating group on C6-position of 3-formylchromones, the yield was decreased (**3m**, **3q** vs. **3a**). Meanwhile, the electron-donating mono-methoxyl group on 3-formylchromones were suitable substrates, affording the corresponding products in excellent yields (**3j**–**3l**, 82–85%). Then, several di-substituted 3-formylchromones were also investigated and gave good results (**3g**, **3n**–**3p**, 40–75%). Interestingly, we found that two electron-donating groups on the benzene ring gave lower yields (**3n**, **3p** vs. **3g**, **3o**). Notably, naphthyl-substituted 3-formylchromones were well-tolerated under the optimized reaction conditions, which afforded the desired products excellent yields up to 95% (**3r**, **3s**). To further explore the substrate scope of this protocol, 2-amino-6,7-dimethoxynaphthalene-1,4-dione was also employed in this reaction, and successfully provided the corresponding product with a good yield (**3t**, 68%). The single crystal structure of **3t** as trifluoroacetic acid salt hydrate was confirmed by X-ray crystallographic analysis (Figure 3).

**Scheme 1:**
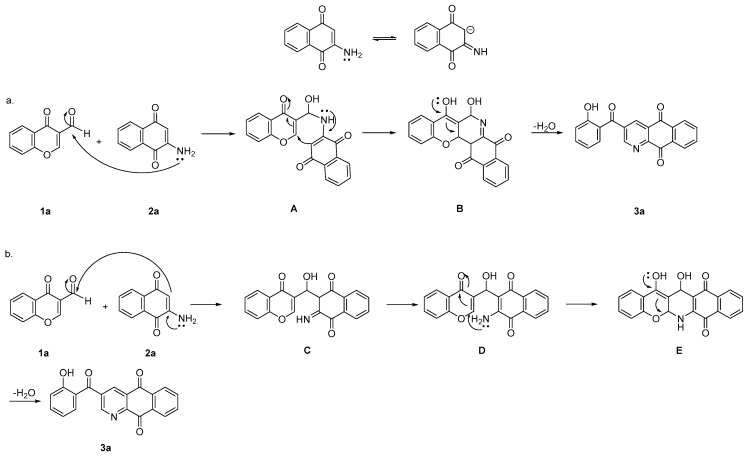
Proposed Mechanisms.

Based on the isomerization of 2-amino-naphthalene-1,4-dione, we proposed two possible mechanisms (Scheme 1). In path A, the reaction initiates nucleophilic attack of 3-formylchromone by the nitrogen atom of **2a** to give intermediate **A**. Then, intramolecular nucleophilic substitution of **A** generates intermediate **B**, which terminates by ring cleavage and aromatization to give the final product **3a**. In path B, the C3-position of **2a** becomes more nucleophilic than the nitrogen atom because of the conjugative effect. Subsequently, like path A, after intramolecular nucleophilic substitution, ring cleavage, and aromatization, the final product **3a** was obtained.

Subsequently, some products were evaluated for anti-proliferative activity against human cervical cancer cell line Hela and colon cancer cell line HT-29 (Table 3). Some compounds displayed moderate to good anti-proliferation activity (such as **3j**, **3l**, **3n**, **3p**, **3q**). Although these compounds were less potent than YCH337 and CA-4 to the Hela cells, compound **3q** exhibited potent antiproliferative activity towards HT-29 cells. 

## 3. Materials and Methods 

### 3.1. General Information

All reagents were purchased from commercial suppliers and used without further purification. The progress of all of the reactions was monitored by thin layer chromatography with standard TLC silica gel plates, and the developed plates were visualized under UV light. All of the compounds were purified by column chromatography. Chromatography was performed on silica gel (100–200 mesh). Nuclear magnetic resonance spectra (^1^H, ^13^C NMR) were recorded on Varian Mercury-300/400 and Varian Mercury-400/500 spectrometers and TFA-d was used as the solvent. NMR peaks were calibrated by reference to standard peaks of TFA at 11.50 ppm for ^1^H and 116.60 and 164.20 ppm for ^13^C. For peak descriptions, the following abbreviations were used: s (singlet), d (doublet), t (triplet), dd (doublet of doublets), td (triplet of doublets), dt (doublet of triplets), pt (pseudo-triplet), ddd (double of doublets of doublets). EI-HRMS were recorded using a Thermo DFS mass spectrometer. The NMR spectra of the obtained compounds and some additional experimental details can be found in the Supplementary Materials.

### 3.2. Crystal Structure Determination of Compound ***3t***

**CCDC 1921125** contains the supplementary crystallographic data for compound **3t**. These data can be obtained free of charge from The Cambridge Crystallographic Data Centre at www.ccdc.cam.ac.uk.

**Crystal Data** for C_24_H_18_F_3_NO_9_ (M = 521.39 g/mol): Orthorhombic, space group P2_1_2_1_2_1_ (no. 19), *a* = 4.8001(2) Å, *b* = 16.6714(8) Å, *c* = 27.6470(14) Å, *V* = 2212.43(18) Å3, *Z* = 4, *T* = 100.15 K, μ(MoKα) = 0.136 mm^-1^, *Dcalc* = 1.565 g/cm^3^, 23769 reflections measured (4.886° ≤ 2Θ ≤ 52.826°), 4503 unique (R_int_ = 0.0380, R_sigma_ = 0.0290), which were used in all calculations. The final R_1_ was 0.0393 (I > 2σ(I)) and *w*R_2_ was 0.1206 (all data).

### 3.3. General Procedure for the Synthesis of Compounds ***1a***–***1t***

To a cooled (0 °C) solution of 2’-hydroxyacetophenone (1 g, 1 eq) in DMF (30 mL), phosphorus oxychloride (5 eq) was added. The mixture was stirred at 64 °C for 4 h until the 2’-hydroxyacetophenone was consumed completely (TLC). The reaction was quenched with glacial water (100 mL), and the mixture was stirred for an additional 30 min. Then, the mixture was extracted three times with dichloromethane (100 mL). The solvent was evaporated in vacuo and the crude product was purified by column chromatography on silica gel.

### 3.4. General Procedure for 2-Amino-naphthalene-1,4-dione(***2a***)

To a cooled (0 °C) solution of hydroxylamine hydrochloride (1.8 g, 2 eq) in EtOH (100 mL), NEt_3_ was added (5.3 mL, 3 eq). To the well stirred mixture, an ethanol solution (20 mL) of 1,4-naphthoquinone (2.0 g, 1 eq) was added dropwise. The mixture was stirred at room temperature for an additional 2 h until 1,4-naphthoquinone was consumed (TLC). The mixture was diluted with water (100 mL), and extracted three times with dichloromethane (30 mL). The organic phase was dried over anhydrous Na_2_SO_4_, concentrated in vacuo, and purified by column chromatography on silica gel.

### 3.5. General Procedure for the Synthesis of Compounds ***3a***–***3t***

To a solution of 2-amino-naphthalene-1,4-dione (0.5 mmol, 1 eq) in AcOH (6 mL), 3-formylchromone (0.6 mmol, 1.2 eq) was added. The resulting reaction mixture was heated to reflux for 4 h. Upon completion (determined by TLC), the reaction mixture was cooled to room temperature, diluted with water (30 mL), and extracted three times with dichloromethane (10 mL). The organic phase was dried over Na_2_SO_4_, concentrated in vacuo, and purified by column chromatography on silica gel. Several compounds were collected by filtration for poor solubility.

*3-(2-Hydroxybenzoyl)benzo[g]quinoline-5,10-dione* (**3a**). Brown solid (136 mg, 83%). ^1^H NMR (500 MHz, TFA-d) δ 9.78 (d, *J* = 5.4 Hz, 1H), 9.71 (d, *J* = 5.0 Hz, 1H), 8.57 (d, *J* = 6.6 Hz, 2H), 8.21–8.10 (m, 2H), 7.81 (t, *J* = 7.3 Hz, 1H), 7.59 (d, *J* = 7.5 Hz, 1H), 7.30 (d, *J* = 8.0 Hz, 1H), 7.19 (t, *J* = 7.1 Hz, 1H). ^13^C NMR (125 MHz, TFA-d) δ 194.56, 179.95, 176.94, 163.62, 148.86, 147.38, 143.09, 142.88, 141.51, 139.48, 138.56, 133.88, 133.21, 132.90, 130.58, 130.43, 123.14, 120.58. HRMS (EI^+^): Calcd. for C_20_H_11_NO_4_ [M]^+^ 329.0688, found: 329.0685.

*3-(5-Fluoro-2-hydroxybenzoyl)benzo[g]quinoline-5,10-dione* (**3b**). Brown solid (139 mg, 80%). ^1^H NMR (500 MHz, TFA-d) δ 9.78 (d, *J* = 2.0 Hz, 1H), 9.72 (d, *J* = 2.3 Hz, 1H), 8.57 (d, *J* = 6.9 Hz, 2H), 8.21–8.11 (m, 2H), 7.56–7.50 (m, 1H), 7.31–7.25 (m, 2H). ^13^C NMR (125 MHz, TFA-d) δ 194.00, 180.36, 177.39, 160.29, 158.43 (d, *J* = 243.2 Hz), 149.43, 147.66, 143.68, 142.90, 139.92, 139.00, 134.29, 133.67, 133.33, 131.01, 130.86, 129.27 (d, *J* = 24.3 Hz), 122.77 (d, *J* = 7.4 Hz), 119.84 (d, *J* = 6.7 Hz), 118.75 (d, *J* = 24.6 Hz). HRMS (EI^+^): Calcd. for C_20_H_10_FNO_4_ [M]^+^ 347.0594, found: 347.0579.

*3-(5-Chloro-2-hydroxybenzoyl)benzo[g]quinoline-5,10-dione* (**3c**). Brown solid (140 mg, 77%). ^1^H NMR (500 MHz, TFA-d) δ 9.78 (d, *J* = 3.8 Hz, 1H), 9.72 (d, *J* = 4.6 Hz, 1H), 8.57 (d, *J* = 7.2 Hz, 2H), 8.22–8.11 (m, 2H), 7.76–7.68 (m, 1H), 7.55 (d, *J* = 2.6 Hz, 1H), 7.27–7.21 (m, 1H). ^13^C NMR (125 MHz, TFA-d) δ 194.01, 180.40, 177.39, 162.61, 149.38, 147.66, 143.68, 142.92, 141.43, 139.92, 139.01, 134.29, 133.72, 133.33, 132.93, 131.02, 130.87, 128.80, 122.62, 120.84. HRMS (EI^+^): Calcd. for C_20_H_10_ClNO_4_ [M]^+^ 363.0298, found: 363.0282.

*3-(5-Bromo-2-hydroxybenzoyl)benzo[g]quinoline-5,1*0-dione (**3d**). Brown solid (184 mg, 90%). ^1^H NMR (400 MHz, TFA-d) δ 9.78 (d, *J* = 2.0 Hz, 1H), 9.72 (d, *J* = 2.0 Hz, 1H), 8.61–8.54 (m, 2H), 8.22–8.10 (m, 2H), 7.86 (dt, *J* = 9.1, 2.0 Hz, 1H), 7.69 (d, *J* = 2.1 Hz, 1H), 7.19 (d, *J* = 9.0 Hz, 1H). ^13^C NMR (125 MHz, TFA-d) δ 193.95, 180.43, 177.39, 163.06, 149.37, 147.66, 144.29, 143.68, 142.93, 139.92, 139.02, 136.07, 134.29, 133.73, 133.34, 131.02, 130.88, 122.85, 121.46, 115.02. HRMS (EI^+^): Calcd. for C_20_H_10_BrNO_4_ [M]^+^ 406.9793, found: 406.9777.

*3-(4-Fluoro-2-hydroxybenzoyl)benzo[g]quinoline-5,10-dione* (**3e**). Brown solid (136 mg, 78%). ^1^H NMR (500 MHz, TFA-d) δ 9.73 (d, *J* = 3.8 Hz, 1H), 9.70 (d, *J* = 4.0 Hz, 1H), 8.57 (t, *J* = 4.3 Hz, 2H), 8.20–8.11 (m, 2H), 7.72–7.56 (m, 1H), 7.00–6.93 (m, 1H), 6.85 (d, *J* = 7.3 Hz, 1H). ^13^C NMR (125 MHz, TFA-d) δ 193.72, 180.39, 177.36, 172.19 (d, *J* = 264.0 Hz), 167.67 (d, *J* = 14.9 Hz), 149.25, 147.52, 143.56, 143.28, 139.91, 139.00, 137.13 (d, *J* = 12.8 Hz), 134.28, 133.66, 133.33, 131.01, 130.86, 117.11, 111.89 (d, *J* = 23.8 Hz), 107.99 (d, *J* = 24.9 Hz). HRMS (EI^+^): Calcd. for C_20_H_10_FNO_4_ [M]^+^ 347.0594, found: 347.0585.

*3-(4-Bromo-2-hydroxybenzoyl)benzo[g]quinoline-5,10-dione* (**3f**). Brown solid (145 mg, 71%). ^1^H NMR (600 MHz, TFA-d) δ 9.75 (d, *J* = 1.7 Hz, 1H), 9.69 (d, *J* = 1.8 Hz, 1H), 8.58 (d, *J* = 1.9 Hz, 1H), 8.57 (d, *J* = 2.0 Hz, 1H), 8.20–8.12 (m, 2H), 7.49 (d, *J* = 1.7 Hz, 1H), 7.44 (d, *J* = 8.6 Hz, 1H), 7.30 (dd, *J* = 8.7, 1.7 Hz, 1H). ^13^C NMR (125 MHz, TFA-d) δ 194.28, 180.40, 177.37, 149.33, 147.59, 143.63, 143.11, 139.91, 139.01, 137.53, 134.82, 134.28, 133.66, 133.34, 131.01, 130.87, 127.06, 124.53, 118.87. HRMS (EI^+^): Calcd. for C_20_H_10_BrNO_4_ [M]^+^ 406.9793, found: 406.9792.

*3-(3,5-Dichloro-2-hydroxybenzoyl)benzo[g]quinoline-5,10-dione* (**3g**). Brown solid (129 mg, 65%). ^1^H NMR (500 MHz, TFA-d) δ 9.79 (d, *J* = 4.5 Hz, 1H), 9.73 (d, *J* = 4.4 Hz, 1H), 8.57 (d, *J* = 7.3 Hz, 2H), 8.24–8.07 (m, 2H), 7.81 (d, *J* = 4.3 Hz, 1H), 7.56 (t, *J* = 3.3 Hz, 1H). ^13^C NMR (125 MHz, TFA-d) δ 192.96, 180.38, 177.39, 156.88, 149.50, 147.75, 143.84, 142.61, 139.92, 139.81, 139.01, 134.26, 133.72, 133.33, 131.69, 131.00, 130.86, 128.86, 126.91, 122.27. HRMS (EI^+^): Calcd. for C_20_H_9_Cl_2_NO_4_ [M]^+^ 396.9909, found: 396.9899.

*3-(2-Hydroxy-5-nitrobenzoyl)benzo[g]quinoline-5,10-dione* (**3h**). Brown solid (133 mg, 71%). ^1^H NMR (600 MHz, TFA-d) δ 9.81 (d, *J* = 1.8 Hz, 1H), 9.78 (d, *J* = 1.9 Hz, 1H), 8.64 (d, *J* = 2.6 Hz, 1H), 8.61 (dd, *J* = 9.5, 2.8 Hz, 1H), 8.60–8.56 (m, 2H), 8.20–8.13 (m, 2H), 7.42 (d, *J* = 9.3 Hz, 1H). ^13^C NMR (125 MHz, TFA-d) δ 193.87, 180.48, 177.45, 169.64, 149.64, 147.52, 144.06, 142.31, 142.26, 139.93, 139.06, 135.01, 134.29, 133.87, 133.37, 131.05, 130.89, 130.87, 122.58, 119.69. HRMS (EI^+^): Calcd. for C_20_H_10_N_2_O_6_ [M]^+^ 374.0539, found: 374.0534.

*3-(5,10-Dioxo-5,10-dihydrobenzo[g]quinoline-3-carbonyl)-4-hydroxybenzonitrile* (**3i**). Brown solid (120 mg, 68%). ^1^H NMR (600 MHz, TFA-d) δ 9.78 (d, *J* = 1.8 Hz, 1H), 9.76 (d, *J* = 1.8 Hz, 1H), 8.57 (td, *J* = 6.5, 5.7, 1.3 Hz, 2H), 8.16 (ddd, *J* = 9.4, 7.3, 1.5 Hz, 2H), 8.13 (d, *J* = 2.0 Hz, 1H), 8.03 (dd, *J* = 8.9, 2.0 Hz, 1H), 7.42 (d, *J* = 8.9 Hz, 1H). ^13^C NMR (125 MHz, TFA-d) δ 193.47, 180.40, 177.43, 167.97, 149.72, 147.48, 143.95, 142.82, 142.31, 139.93, 139.78, 139.03, 134.27, 133.71, 133.34, 131.03, 130.87, 122.88, 121.05, 104.89. HRMS (EI^+^): Calcd. for C_21_H_10_N_2_O_4_ [M]^+^ 354.0641, found: 354.0636.

*3-(2-Hydroxy-5-methoxybenzoyl)benzo[g]quinoline-5,10-dione* (**3j**). Brown solid (153 mg, 85%). ^1^H NMR (600 MHz, TFA-d) δ 9.79 (d, *J* = 1.7 Hz, 1H), 9.73 (d, *J* = 1.8 Hz, 1H), 8.60–8.56 (m, 2H), 8.18 (td, *J* = 7.6, 1.6 Hz, 1H), 8.14 (td, *J* = 7.6, 1.6 Hz, 1H), 7.54 (dd, *J* = 9.3, 3.0 Hz, 1H), 7.34 (d, *J* = 3.0 Hz, 1H), 7.30 (d, *J* = 9.2 Hz, 1H), 3.96 (s, 3H). ^13^C NMR (125 MHz, TFA-d) δ 194.45, 180.40, 177.37, 158.75, 154.35, 149.35, 147.91, 143.43, 143.04, 139.93, 138.98, 134.30, 133.68, 133.30, 131.04, 130.85, 128.34, 122.32, 120.31, 119.65, 58.52. HRMS (EI^+^): Calcd. for C_21_H_13_NO_5_ [M]^+^ 359.0794, found: 359.0786.

*3-(2-Hydroxy-4-methoxybenzoyl)benzo[g]quinoline-5,10-dione* (**3k**). Brown solid (150 mg, 84%). ^1^H NMR (500 MHz, TFA-d) δ 9.72 (d, *J* = 1.7 Hz, 1H), 9.67 (d, *J* = 1.8 Hz, 1H), 8.59–8.55 (m, 2H), 8.15 (td, *J* = 7.1, 1.6 Hz, 2H), 7.47 (d, *J* = 9.1 Hz, 1H), 6.80 (d, *J* = 2.4 Hz, 1H), 6.73 (dd, *J* = 9.1, 2.4 Hz, 1H), 4.03 (s, 3H). ^13^C NMR (125 MHz, TFA-d) δ 192.61, 180.44, 177.36, 172.06, 168.41, 149.00, 147.55, 143.62, 143.32, 139.90, 138.99, 136.50, 134.30, 133.65, 133.32, 131.01, 130.86, 114.14, 112.71, 103.94, 57.51. HRMS (EI^+^): Calcd. for C_21_H_13_NO_5_ [M]^+^ 359.0794, found: 359.0780.

*3-(2-Hydroxy-6-methoxybenzoyl)benzo[g]quinoline-5,10-dione* (**3l**). Brown solid (148 mg, 82%). ^1^H NMR (500 MHz, TFA-d) δ 9.63 (s, 1H), 9.54 (s, 1H), 8.56 (d, *J* = 7.3 Hz, 2H), 8.19–8.09 (m, 2H), 7.76–7.67 (m, 1H), 6.89 (d, *J* = 8.4 Hz, 1H), 6.70 (d, *J* = 8.3 Hz, 1H), 3.68 (s, 3H). ^13^C NMR (125 MHz, TFA-d) δ 193.46, 180.58, 177.39, 162.57, 148.24, 146.85, 146.57, 142.65, 142.54, 139.83, 138.92, 134.29, 133.38, 133.29, 130.96, 130.78, 115.89, 111.61, 105.59, 57.17. HRMS (EI^+^): Calcd. for C_21_H_13_NO_5_ [M]^+^ 359.0794, found: 359.0786.

*3-(2-Hydroxy-5-methylbenzoyl)benzo[g]quinoline-5,10-dione* (**3m**). Brown solid (127 mg, 74%). ^1^H NMR (400 MHz, TFA-d) δ 9.77 (s, 1H), 9.71 (s, 1H), 8.58 (d, *J* = 2.8 Hz, 1H), 8.57 (d, *J* = 2.5 Hz, 1H), 8.22–8.09 (m, 2H), 7.66 (d, *J* = 8.6 Hz, 1H), 7.35 (s, 1H), 7.21 (dd, *J* = 8.6, 2.0 Hz, 1H), 2.35 (s, 3H). ^13^C NMR (125 MHz, TFA-d) δ 194.98, 180.42, 177.36, 161.82, 149.24, 147.73, 143.45, 143.27, 139.91, 139.00, 134.41, 134.30, 133.68, 133.62, 133.33, 131.01, 130.86, 120.78, 119.86, 20.74. HRMS (EI^+^): Calcd. for C_21_H_13_NO_4_ [M]^+^ 343.0845, found: 343.0838.

*3-(2-Hydroxy-4,5-dimethylbenzoyl)benzo[g]quinoline-5,10-dione* (**3n**). Brown solid (97 mg, 54%). ^1^H NMR (400 MHz, TFA-d) δ 9.77 (d, *J* = 2.3 Hz, 1H), 9.70 (d, *J* = 2.3 Hz, 1H), 8.58 (dt, *J* = 7.3, 2.2 Hz, 2H), 8.22–8.11 (m, 2H), 7.28 (s, 1H), 7.12 (s, 1H), 2.45 (d, *J* = 2.8 Hz, 3H), 2.27 (d, *J* = 2.7 Hz, 3H). ^13^C NMR (125 MHz, TFA-d) δ 194.28, 180.43, 177.35, 162.50, 155.37, 149.11, 147.70, 143.60, 143.37, 139.90, 138.99, 134.30, 134.07, 133.67, 133.61, 133.32, 131.00, 130.85, 121.44, 118.02, 21.46, 19.28. HRMS (EI^+^): Calcd. for C_22_H_15_NO_4_ [M]^+^ 357.1001, found: 357.1001.

*3-(5-Chloro-2-hydroxy-4-methylbenzoyl)benzo[g]quinoline-5,10-dione* (**3o**). Brown solid (142 mg, 75%). ^1^H NMR (400 MHz, TFA-d) δ 9.78 (d, *J* = 1.9 Hz, 1H), 9.71 (d, *J* = 2.0 Hz, 1H), 8.60–8.54 (m, 2H), 8.21 − 8.11 (m, 2H), 7.52 (s, 1H), 7.20 (s, 1H), 2.53 (s, 3H). ^13^C NMR (125 MHz, TFA-d) δ 193.58, 180.45, 177.38, 162.69, 153.15, 149.28, 147.63, 143.59, 143.10, 139.92, 139.02, 134.29, 133.71, 133.42, 133.33, 131.02, 130.87, 129.47, 122.92, 119.03, 21.72. HRMS (EI^+^): Calcd. for C_21_H_12_ClNO_4_ [M]^+^ 377.0455, found: 377.0447.

*3-(2-Hydroxy-4,5-dimethoxybenzoyl)benzo[g]quinoline-5,10-dione* (**3p**). Brown solid (78 mg, 40%). ^1^H NMR (600 MHz, TFA-d) δ 9.76 (s, 1H), 9.72 (s, 1H), 8.61–8.54 (m, 2H), 8.17 (t, *J* = 7.4 Hz, 1H), 8.14 (t, *J* = 7.4 Hz, 1H), 7.10 (s, 1H), 6.88 (s, 1H), 4.11 (s, 3H), 3.90 (s, 3H). ^13^C NMR (125 MHz, TFA-d) δ 192.10, 180.42, 177.39, 164.78, 162.56, 149.26, 147.37, 144.83, 143.67, 143.35, 139.87, 138.97, 134.27, 133.65, 133.34, 130.99, 130.84, 116.51, 112.44, 103.41, 59.17, 57.98. HRMS (EI^+^): Calcd. for C_22_H_15_NO_6_ [M]^+^ 389.0899, found: 389.0891.

*3-(2-Hydroxy-5-morpholinobenzoyl)benzo[g]quinoline-5,10-dione* (**3q**). Brown solid (142 mg, 69%). ^1^H NMR (400 MHz, TFA-d) δ 9.81 (s, 1H), 9.76 (s, 1H), 8.59 (d, *J* = 6.5 Hz, 2H), 8.22–8.12 (m, 3H), 8.05 (d, *J* = 9.4 Hz, 1H), 7.51 (d, *J* = 9.3 Hz, 1H), 4.42 (s, 4H), 4.00 (s, 4H). ^13^C NMR (125 MHz, TFA-d) δ 192.44, 180.52, 177.44, 149.90, 147.56, 143.92, 142.24, 139.97, 139.06, 134.91, 134.26, 133.64, 133.33, 131.33, 131.14, 130.89, 127.20, 124.13, 121.87, 66.56, 58.28. HRMS (EI^+^): Calcd. for C_24_H_18_N_2_O_5_ [M]^+^ 414.1216, found: 414.1212.

*3-(2-Hydroxy-1-naphthoyl)benzo[g]quinoline-5,10-dione* (**3r**). Brown solid (181 mg, 95%). ^1^H NMR (600 MHz, TFA-d) δ 9.75 (d, *J* = 1.8 Hz, 1H), 9.62 (d, *J* = 1.8 Hz, 1H), 8.53 (dd, *J* = 7.4, 1.6 Hz, 1H), 8.51 (dd, *J* = 7.4, 1.6 Hz, 1H), 8.16–8.09 (m, 3H), 7.90 (dd, *J* = 7.1, 2.2 Hz, 1H), 7.67 (d, *J* = 8.7 Hz, 1H), 7.46 (tt, *J* = 7.1, 5.4 Hz, 2H), 7.27 (d, *J* = 9.0 Hz, 1H). ^13^C NMR (150 MHz, TFA-d) δ 193.30, 180.41, 177.33, 161.62, 149.80, 148.43, 144.72, 143.24, 141.23, 139.88, 138.92, 134.23, 133.77, 133.41, 133.25, 131.67, 131.59, 131.52, 130.97, 130.78, 127.67, 125.92, 119.75, 115.50. HRMS (EI^+^): Calcd. for C_24_H_13_NO_4_ [M]^+^ 379.0845, found: 379.0812.

*3-(1-Hydroxy-2-naphthoyl)benzo[g]quinoline-5,10-dione* (**3s**). Brown solid (175 mg, 92%). ^1^H NMR (400 MHz, TFA-d) δ 9.81 (s, 1H), 9.74 (s, 1H), 8.57 (t, *J* = 6.6 Hz, 3H), 8.21– 8.09 (m, 2H), 7.83 (d, *J* = 6.3 Hz, 2H), 7.68 (t, *J* = 7.2 Hz, 1H), 7.40 (d, *J* = 8.7 Hz, 1H), 7.30 (d, *J* = 8.5 Hz, 1H). ^13^C NMR (125 MHz, TFA-d) δ 193.82, 180.41, 177.37, 168.46, 149.07, 147.72, 143.85, 143.23, 141.06, 139.90, 138.98, 134.96, 134.29, 133.60, 133.32, 131.00, 130.85, 129.93, 129.52, 126.89, 126.77, 125.74, 123.05, 113.45. HRMS (EI^+^): Calcd. for C_24_H_13_NO_4_ [M]^+^ 379.0845, found: 379.0829.

*3-(2-Hydroxybenzoyl)-7,8-dimethoxybenzo[g]quinoline-5,10-dione* (**3t**). Brown solid (132 mg, 68%). ^1^H NMR (500 MHz, TFA-d ) δ 9.71 (s, 1H), 9.63 (s, 1H), 8.04 (s, 1H), 8.03 (s, 1H), 7.81 (t, *J* = 8.0 Hz, 1H), 7.58 (d, *J* = 8.0 Hz, 1H), 7.30 (d, *J* = 8.5 Hz, 1H), 7.18 (t, *J* = 7.7 Hz, 1H), 4.25 (d, *J* = 3.3 Hz, 6H). ^13^C NMR (125 MHz, TFA-d) δ 195.04, 179.45, 175.96, 164.04, 158.82, 157.82, 148.49, 147.32, 143.89, 143.09, 141.92, 134.31, 133.64, 130.31, 128.93, 123.55, 121.00, 120.03, 112.03, 111.60, 58.36. HRMS (EI^+^): Calcd. for C_22_H_15_NO_6_ [M]^+^ 389.0899, found: 389.0885.

## 4. Conclusions

We have developed a facile, efficient, and mild synthetic strategy to assemble 3-(2-hydroxybenzoyl)-1-aza-anthraquinone derivatives from 3-formylchromones and 2-amino-naphthalene-1,4-dione. Most of the substrates bearing diverse substituents worked well with this procedure, and a great variety of 3-(2-hydroxybenzoyl)-1-aza-anthraquinone derivatives were successfully obtained in moderate to excellent yields, which are potentially useful skeletons in pharmaceutical discovery research. Moreover, this reaction could proceed without metal catalysis and be insensitive to air and moisture.

## Figures and Tables

**Table 1:**
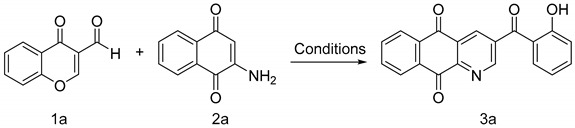
Optimization of the reaction conditions ^a^.

Entry	Ratio (2a:1a)	Solvent	Temp (°C)	Time (h)	Yield ^b^ (%)
1	1:1.1	AcOH	80	24	35
2	1:1.1	AcOH	100	19	67
3	1:1.1	AcOH	Reflux	4	76
4	1:1.1	DMF	120	24	n.d. ^c^
5	1:1.1	DMSO	120	24	32
6	1:1.1	Propionic acid	120	19	70
7	1:1.1	1,4-dioxane	Reflux	24	n.d. ^c^
8	1:1.1	AcOH:Et_3_N (1:1)	100	24	48
9	1:1	AcOH	Reflux	4	71
10	1:1.2	AcOH	Reflux	4	83
11	1:1.3	AcOH	Reflux	4	70
12	1:1.5	AcOH	Reflux	4	77

^a^ Reaction conditions: **2** (0.50 mmol, 87 mg), solvent (6.0 mL). ^b^ Isolated yield. ^c^ n.d. = not detected.

**Table 2:** Scope of the target compounds ^a, b^.

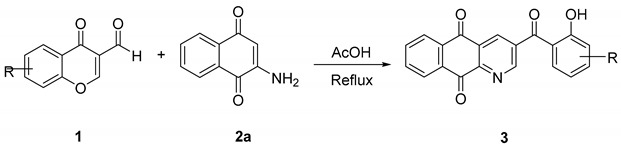
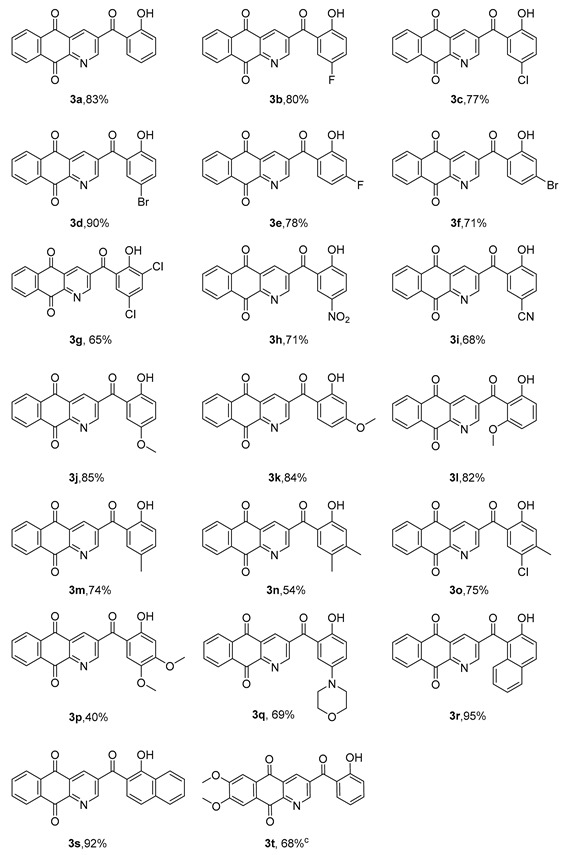

^a^ Reaction conditions: **1** (0.6 mmol), **2** (0.5 mmol), and AcOH (6.0 mL) in air refluxed for 4 h. ^b^ Isolated yields. ^c^ **1a** (0.6 mmol), 2-amino-6,7-dimethoxynaphthalene-1,4-dione (0.5 mmol) was used.

**Table 3:** Anti-proliferation activity of selected compounds ^a.^

Compd	IC_50_ [μM] ^b,c^	
	Hela	HT-29
**3q**	1.99	0.018
**3p**	4.69	2.26
**3j**	7.98	4.15
**3n**	-	4.41
**3l**	12.81	1.27
**CA-4**	0.78 (nM)	0.74
**YCH337**	0.028	0.063

^a ^The experiments were assessed by SRB assay. ^b^ Exposure time: 72 h. ^c ^The average IC_50_ values were determined by at least three independent tests.

## Supplementary Materials

The NMR spectra of the obtained compounds and some additional experimental details are available online.
