# Supplementary material for: One Pot and Metal-Free Approach to 3-(2-Hydroxybenzoyl)-1-aza-anthraquinones
Source: Molecules. 2019 Aug 20;24(16):3017. doi: 10.3390/molecules24163017 (PMC6720541; doi:10.3390/molecules24163017)
Supplement: Supplementary file 1 [file molecules-24-03017-s001.pdf]

Supporting Information

One pot and metal-free approach to 3-(2-hydroxybenzoyl)-1-azaanthraquinones

Jiaqi Yuan,[ab] Qian He,[a] Shanshan Song,[a] Xiaofei Zhang*[a] Zehong Miao,[a] and Chunhao Yang*[a]

[a]State Key Laboratory of Drug Research, Shanghai Institute of Materia Medica, Chinese Academy of Science, 555 Zu Chong Zhi Road, Shanghai 201203, China

[b]School of Pharmacy, University of Chinese Academy of Sciences, No. 19A Yuquan Road, Beijing 100049, China.

**E-mail: chyang@simm.ac.cn(C.Yang); xiaofeizhang@simm.ac.cn(X. Zhang) .*

Table of Contents

I. General information......2
II. General experimental procedures
1. General procedure for the synthesis of compounds **1a-1t**......3
2. General procedure for 2-amino-naphthalene-1,4-dione......3
3. General procedure for the synthesis of compounds **3a-3t**......3
III. X-Ray Analysis......5
IV. General procedure of biology......6
V. Compound characterizations......7
VI. ^{1}H and ^{13}C NMR spectra......13

I. General information

All reagents were purchased from commercial suppliers and used without further purification. The progress of all of the reactions was monitored by thin layer chromatography with standard TLC silica gel plates, and the developed plates were visualized under UV light. All of the compounds were purified by column chromatography. Chromatography was performed on silica gel (100–200 mesh). Nuclear magnetic resonance spectra (^{1}H, ^{13}C NMR) were recorded on Varian Mercury- 300/400 and Varian Mercury-400/500 spectrometers and TFA-d was used as the solvent. NMR peaks were calibrated by reference to standard peaks of TFA at 11.50 ppm for ^{1}H and 116.60 and 164.20 ppm for ^{13}C. For peak descriptions, the following abbreviations have been used: s (singlet), d (doublet), t (triplet), dd (doublet of doublets), td (triplet of doublets), dt (doublet of triplets), pt (pseudo-triplet), ddd (double of doublets of doublets). EI-HRMS were recorded using Thermo DFS mass spectrometer.

II. General experimental methods

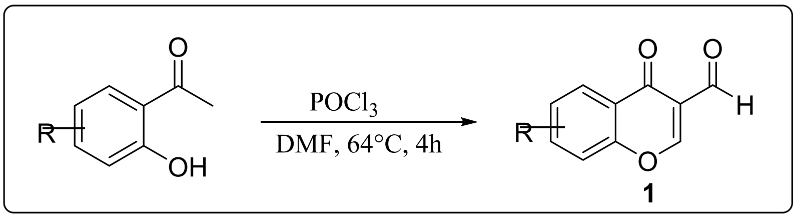

1. General procedure for the synthesis of compounds 1a-1t: To a cooled (0°C) solution of 2'-hydroxyacetophenone (1 g, 1 eq) in DMF (30 mL) was added phosphorus oxychloride (5 eq). The mixture stirred at 64 °C for 4 h until the 2'-hydroxyacetophenone consumed completely (TLC). The reaction was quenched with glacial water (100 mL), and the mixture stirred for an additional 30 mins. Then the mixture was extracted three times with dichloromethane (100 mL). And then the solvent was evaporated *in vacuo* and the crude product was purified by column chromatography on silica gel.

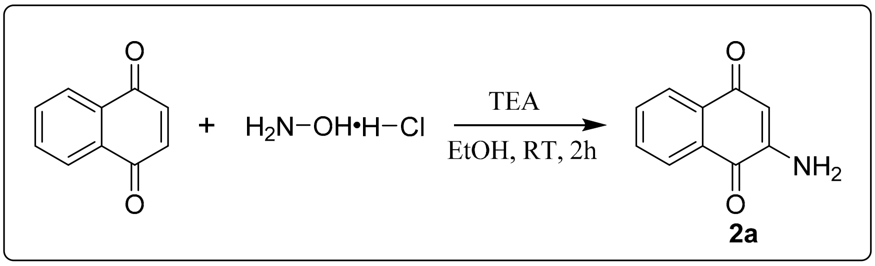

2. General procedure for 2-amino-naphthalene-1,4-dione: To a cooled (0 °C) solution of hydroxylamine hydrochloride (1.8 g, 2 eq) in EtOH (100 mL), NEt_3 was added (5.3 mL, 3 eq). To the well stirred mixture was added dropwise an ethanol solution (20 mL) of 1,4-naphthoquinone (2.0 g, 1 eq). The mixture stirred at room temperature for an additional 2 hours until 1,4-naphthoquinone consumed (TLC). The mixture was diluted with water (100 mL), and extracted three times with dichloromethane (30 mL). The organic phase was dried over anhydrous Na_2SO_4, concentrated *in vacuo*, and purified by column chromatography on silica gel.

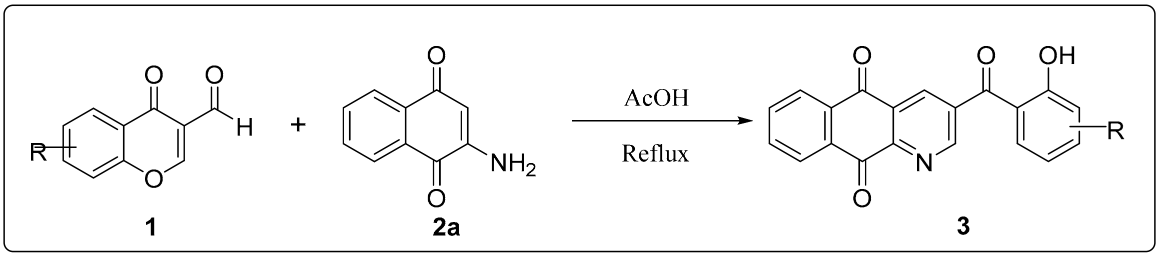

3. General procedure for the synthesis of compounds 3a-3t: To a solution of 2-amino-naphthalene-1,4-dione (0.5 mmol, 1 eq) in AcOH (6 mL), 3-formylchromone (0.6 mmol, 1.2 eq) was added. The resulting reaction mixture was heated to reflux for

4 hours. Upon completion (determined by TLC), the reaction mixture was cooled to room temperature, diluted with water (30 mL), and extracted three times with dichloromethane (10 mL). The organic phase was dried over Na_2SO_4, concentrated *in vacuo*, and purified by column chromatography on silica gel. Several compounds were collected by filtration for poor solubility.

III. X-Ray Analysis

CCDC 1921125 contains the supplementary crystallographic data for compound **3t**. These data can be obtained free of charge from The Cambridge Crystallographic Data Centre at www.ccdc.cam.ac.uk.

Crystal structure determination of compound 3t:

Crystal Data for $C_{24}H_{18}F_3NO_9$ (M =521.39 g/mol): orthorhombic, space group $P2_12_12_1$ (no. 19), a = 4.8001(2) Å, b = 16.6714(8) Å, c = 27.6470(14) Å, V = 2212.43(18) Å3, Z = 4, T = 100.15 K, μ(MoKα) = 0.136 mm^{-1}, $Dcalc$ = 1.565 g/cm^3, 23769 reflections measured (4.886° ≤ 2Θ ≤ 52.826°), 4503 unique (R_{int} = 0.0380, R_{sigma} = 0.0290) which were used in all calculations. The final R_1 was 0.0393 (I > 2σ(I)) and wR_2 was 0.1206 (all data).

Table 1 Crystal data and structure refinement for compound **3t**.

Identification code	compound **3t**
Empirical formula	$C_{24}H_{18}F_3NO_9$
Formula weight	521.39
Temperature/K	100.15
Crystal system	orthorhombic
Space group	$P2_12_12_1$
a/Å	4.8001(2)
b/Å	16.6714(8)
c/Å	27.6470(14)
α/°	90
β/°	90
γ/°	90
Volume/Å^3	2212.43(18)
Z	4
ρ_{calc}g/cm^3	1.565
μ/mm^{-1}	0.136
F(000)	1072.0
Crystal size/mm^3	0.15 × 0.08 × 0.03
Radiation	MoKα (λ = 0.71073)
2Θ range for data collection/°	4.886 to 52.826
Index ranges	-6 ≤ h ≤ 5, -20 ≤ k ≤ 20, -34 ≤ l ≤ 34
Reflections collected	23769
Independent reflections	4503 [R_{int} = 0.0380, R_{sigma} = 0.0290]
Data/restraints/parameters	4503/0/345
Goodness-of-fit on F^2	0.958
Final R indexes [I>=2σ (I)]	R_1 = 0.0393, wR_2 = 0.1155
Final R indexes [all data]	R_1 = 0.0454, wR_2 = 0.1206
Largest diff. peak/hole / e Å^{-3}	0.21/-0.24
Flack parameter	-0.3(10)

IV. General procedure of biology:

The cytotoxicity of the tested compounds to HeLa cells and human colon cancer cell line HT-29 was assayed using the SRB method. YCH337 and Combretastatin A4 (CA4) were used as positive controls to evaluate the inhibitory effect of the tested compounds. The sulforhodamine B (SRB) assay: Cells were seeded into 96-well plates on day 0 and exposed to 2-fold serial drug dilutions on day 1. On day 4, the cells were fixed by adding 10% pre-cooled trichloroacetic acid. After 1 h at 4 ℃, the plates were washed with distilled water, dried, and then stained with SRB (Sigma, MO) in 1% acetic acid. SRB in the cells was dissolved in 10 mM Tris-HCl and was measured at 515 nm using spectra-MAX190 (Molecular Devices, CA). The cell proliferation inhibition rate was calculated as: proliferation inhibition (%) = [1 − (A515treated/A515control)] × 100%.

V. Characterization Data of Compounds

3-(2-hydroxybenzoyl)benzo[*g*]quinoline-5,10-dione (**3a**)

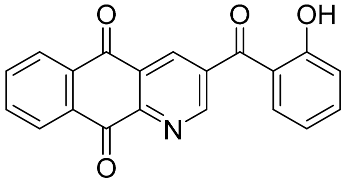

Brown solid (136 mg, 83%). ^{1}H NMR (500 MHz, TFA-d) δ 9.78 (d, *J* = 5.4 Hz, 1H), 9.71 (d, *J* = 5.0 Hz, 1H), 8.57 (d, *J* = 6.6 Hz, 2H), 8.21 – 8.10 (m, 2H), 7.81 (t, *J* = 7.3 Hz, 1H), 7.59 (d, *J* = 7.5 Hz, 1H), 7.30 (d, *J* = 8.0 Hz, 1H), 7.19 (t, *J* = 7.1 Hz, 1H). ^{13}C NMR (125 MHz, TFA-d) δ 194.56, 179.95, 176.94, 163.62, 148.86, 147.38, 143.09, 142.88, 141.51, 139.48, 138.56, 133.88, 133.21, 132.90, 130.58, 130.43, 123.14, 120.58. HRMS (EI^{+}): calcd for $C_{20}H_{11}NO_4$ $[M]^{+}$ 329.0688, found: 329.0685.

3-(5-fluoro-2-hydroxybenzoyl)benzo[*g*]quinoline-5,10-dione (**3b**)

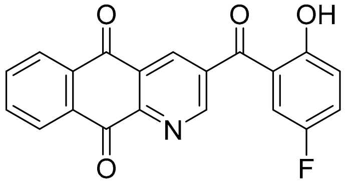

Brown solid (139 mg, 80%). ^{1}H NMR (500 MHz, TFA-d) δ 9.78 (d, *J* = 2.0 Hz, 1H), 9.72 (d, *J* = 2.3 Hz, 1H), 8.57 (d, *J* = 6.9 Hz, 2H), 8.21 – 8.11 (m, 2H), 7.56 – 7.50 (m, 1H), 7.31 – 7.25 (m, 2H). ^{13}C NMR (125 MHz, TFA-d) δ 194.00, 180.36, 177.39, 160.29, 158.43 (d, J = 243.2 Hz), 149.43, 147.66, 143.68, 142.90, 139.92, 139.00, 134.29, 133.67, 133.33, 131.01, 130.86, 129.27 (d, *J* = 24.3 Hz), 122.77 (d, *J* = 7.4 Hz), 119.84 (d, *J* = 6.7 Hz), 118.75 (d, *J* = 24.6 Hz). HRMS (EI^{+}): calcd for $C_{20}H_{10}FNO_4$ $[M]^{+}$ 347.0594, found: 347.0579.

3-(5-chloro-2-hydroxybenzoyl)benzo[*g*]quinoline-5,10-dione (**3c**)

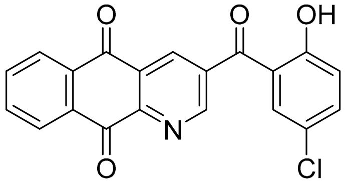

Brown solid (140 mg, 77%). ^{1}H NMR (500 MHz, TFA-d) δ 9.78 (d, *J* = 3.8 Hz, 1H), 9.72 (d, *J* = 4.6 Hz, 1H), 8.57 (d, *J* = 7.2 Hz, 2H), 8.22 – 8.11 (m, 2H), 7.76 – 7.68 (m, 1H), 7.55 (d, *J* = 2.6 Hz, 1H), 7.27 – 7.21 (m, 1H). ^{13}C NMR (125 MHz, TFA-d) δ 194.01, 180.40, 177.39, 162.61, 149.38, 147.66, 143.68, 142.92, 141.43, 139.92, 139.01, 134.29, 133.72, 133.33, 132.93, 131.02, 130.87, 128.80, 122.62, 120.84. HRMS (EI^{+}): calcd for $C_{20}H_{10}ClNO_4$ $[M]^{+}$ 363.0298, found: 363.0282.

3-(5-bromo-2-hydroxybenzoyl)benzo[*g*]quinoline-5,10-dione (**3d**)

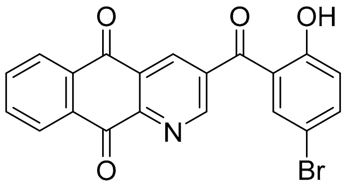

Brown solid (184 mg, 90%). ^{1}H NMR (400 MHz, TFA-d) δ 9.78 (d, *J* = 2.0 Hz, 1H), 9.72 (d, *J* = 2.0 Hz, 1H), 8.61 – 8.54 (m, 2H), 8.22 – 8.10 (m, 2H), 7.86 (dt, *J* = 9.1, 2.0 Hz, 1H), 7.69 (d, *J* = 2.1 Hz, 1H), 7.19 (d, *J* = 9.0 Hz, 1H). ^{13}C NMR (125 MHz, TFA-d) δ 193.95, 180.43, 177.39, 163.06, 149.37, 147.66, 144.29, 143.68, 142.93, 139.92, 139.02, 136.07, 134.29, 133.73, 133.34, 131.02, 130.88, 122.85, 121.46, 115.02. HRMS (EI^{+}): calcd for $C_{20}H_{10}BrNO_4$ $[M]^+$ 406.9793, found: 406.9777.

3-(4-fluoro-2-hydroxybenzoyl)benzo[*g*]quinoline-5,10-dione (**3e**)

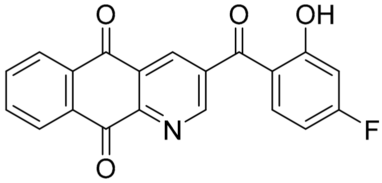

Brown solid (136 mg, 78%). ^{1}H NMR (500 MHz, TFA-d) δ 9.73 (d, *J* = 3.8 Hz, 1H), 9.70 (d, *J* = 4.0 Hz, 1H), 8.57 (t, *J* = 4.3 Hz, 2H), 8.20 – 8.11 (m, 2H), 7.72 – 7.56 (m, 1H), 7.00 – 6.93 (m, 1H), 6.85 (d, *J* = 7.3 Hz, 1H). ^{13}C NMR (125 MHz, TFA-d) δ 193.72, 180.39, 177.36, 172.19 (d, *J* = 264.0 Hz), 167.67 (d, *J* = 14.9 Hz), 149.25, 147.52, 143.56, 143.28, 139.91, 139.00, 137.13 (d, *J* = 12.8 Hz), 134.28, 133.66, 133.33, 131.01, 130.86, 117.11, 111.89 (d, *J* = 23.8 Hz), 107.99 (d, *J* = 24.9 Hz). HRMS (EI^{+}): calcd for $C_{20}H_{10}FNO_4$ $[M]^+$ 347.0594, found: 347.0585.

3-(4-bromo-2-hydroxybenzoyl)benzo[*g*]quinoline-5,10-dione (**3f**)

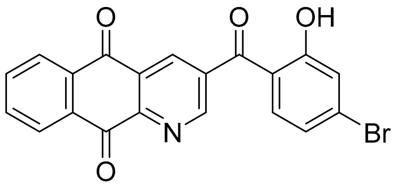

Brown solid (145 mg, 71%). ^{1}H NMR (600 MHz, TFA-d) δ 9.75 (d, *J* = 1.7 Hz, 1H), 9.69 (d, *J* = 1.8 Hz, 1H), 8.58 (d, *J* = 1.9 Hz, 1H), 8.57 (d, *J* = 2.0 Hz, 1H), 8.20 – 8.12 (m, 2H), 7.49 (d, *J* = 1.7 Hz, 1H), 7.44 (d, *J* = 8.6 Hz, 1H), 7.30 (dd, *J* = 8.7, 1.7 Hz, 1H). ^{13}C NMR (125 MHz, TFA-d) δ 194.28, 180.40, 177.37, 149.33, 147.59, 143.63, 143.11, 139.91, 139.01, 137.53, 134.82, 134.28, 133.66, 133.34, 131.01, 130.87, 127.06, 124.53, 118.87. HRMS (EI^{+}): calcd for $C_{20}H_{10}BrNO_4$ $[M]^+$ 406.9793, found: 406.9792.

3-(3,5-dichloro-2-hydroxybenzoyl)benzo[*g*]quinoline-5,10-dione (**3g**)

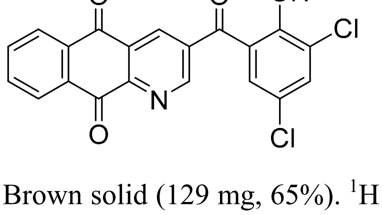

Brown solid (129 mg, 65%). ^{1}H NMR (500 MHz, TFA-d) δ 9.79 (d, *J* = 4.5 Hz, 1H), 9.73 (d, *J* = 4.4 Hz, 1H), 8.57 (d, *J* = 7.3 Hz, 2H), 8.24 – 8.07 (m, 2H), 7.81 (d, *J* = 4.3 Hz, 1H), 7.56 (t,

J = 3.3 Hz, 1H). ^{13}C NMR (125 MHz, TFA-d) δ 192.96, 180.38, 177.39, 156.88, 149.50, 147.75, 143.84, 142.61, 139.92, 139.81, 139.01, 134.26, 133.72, 133.33, 131.69, 131.00, 130.86, 128.86, 126.91, 122.27. HRMS (EI$^+$): calcd for $C_{20}H_9Cl_2NO_4$ [M]$^+$ 396.9909, found: 396.9899.

3-(2-hydroxy-5-nitrobenzoyl)benzo[*g*]quinoline-5,10-dione (**3h**)

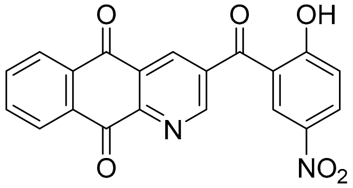

Brown solid (133 mg, 71%). ^{1}H NMR (600 MHz, TFA-d) δ 9.81 (d, J = 1.8 Hz, 1H), 9.78 (d, J = 1.9 Hz, 1H), 8.64 (d, J = 2.6 Hz, 1H), 8.61 (dd, J = 9.5, 2.8 Hz, 1H), 8.60 – 8.56 (m, 2H), 8.20 – 8.13 (m, 2H), 7.42 (d, J = 9.3 Hz, 1H). ^{13}C NMR (125 MHz, TFA-d) δ 193.87, 180.48, 177.45, 169.64, 149.64, 147.52, 144.06, 142.31, 142.26, 139.93, 139.06, 135.01, 134.29, 133.87, 133.37, 131.05, 130.89, 130.87, 122.58, 119.69. HRMS (EI$^+$): calcd for $C_{20}H_{10}N_2O_6$ [M]$^+$ 374.0539, found: 374.0534.

3-(5,10-dioxo-5,10-dihydrobenzo[*g*]quinoline-3-carbonyl)-4-hydroxybenzonitrile (**3i**)

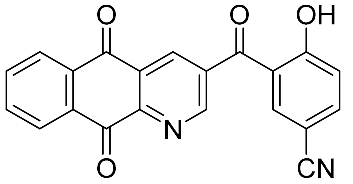

Brown solid (120 mg, 68%). ^{1}H NMR (600 MHz, TFA-d) δ 9.78 (d, J = 1.8 Hz, 1H), 9.76 (d, J = 1.8 Hz, 1H), 8.57 (td, J = 6.5, 5.7, 1.3 Hz, 2H), 8.16 (ddd, J = 9.4, 7.3, 1.5 Hz, 2H), 8.13 (d, J = 2.0 Hz, 1H), 8.03 (dd, J = 8.9, 2.0 Hz, 1H), 7.42 (d, J = 8.9 Hz, 1H). ^{13}C NMR (125 MHz, TFA-d) δ 193.47, 180.40, 177.43, 167.97, 149.72, 147.48, 143.95, 142.82, 142.31, 139.93, 139.78, 139.03, 134.27, 133.71, 133.34, 131.03, 130.87, 122.88, 121.05, 104.89. HRMS (EI$^+$): calcd for $C_{21}H_{10}N_2O_4$ [M]$^+$ 354.0641, found: 354.0636.

3-(2-hydroxy-5-methoxybenzoyl)benzo[*g*]quinoline-5,10-dione (**3j**)

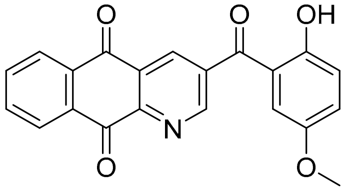

Brown solid (153 mg, 85%). ^{1}H NMR (600 MHz, TFA-d) δ 9.79 (d, J = 1.7 Hz, 1H), 9.73 (d, J = 1.8 Hz, 1H), 8.60 – 8.56 (m, 2H), 8.18 (td, J = 7.6, 1.6 Hz, 1H), 8.14 (td, J = 7.6, 1.6 Hz, 1H), 7.54 (dd, J = 9.3, 3.0 Hz, 1H), 7.34 (d, J = 3.0 Hz, 1H), 7.30 (d, J = 9.2 Hz, 1H), 3.96 (s, 3H). ^{13}C NMR (125 MHz, TFA-d) δ 194.45, 180.40, 177.37, 158.75, 154.35, 149.35, 147.91, 143.43, 143.04, 139.93, 138.98, 134.30, 133.68, 133.30, 131.04, 130.85, 128.34, 122.32, 120.31, 119.65, 58.52. HRMS (EI$^+$): calcd for $C_{21}H_{13}NO_5$ [M]$^+$ 359.0794, found: 359.0786.

3-(2-hydroxy-4-methoxybenzoyl)benzo[*g*]quinoline-5,10-dione (**3k**)

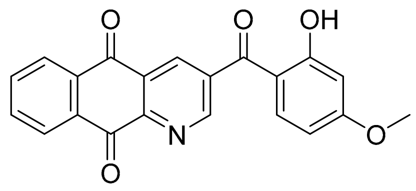

Brown solid (150 mg, 84%). ^{1}H NMR (500 MHz, TFA-d) δ 9.72 (d, J = 1.7 Hz, 1H), 9.67 (d, J = 1.8 Hz, 1H), 8.59 – 8.55 (m, 2H), 8.15 (td, J = 7.1, 1.6 Hz, 2H), 7.47 (d, J = 9.1 Hz, 1H), 6.80 (d, J = 2.4 Hz, 1H), 6.73 (dd, J = 9.1, 2.4 Hz, 1H), 4.03 (s, 3H). ^{13}C NMR (125 MHz, TFA-d) δ 192.61, 180.44, 177.36, 172.06, 168.41, 149.00, 147.55, 143.62, 143.32, 139.90, 138.99, 136.50, 134.30, 133.65, 133.32, 131.01, 130.86, 114.14, 112.71, 103.94, 57.51. HRMS (EI$^+$): calcd for $C_{21}H_{13}NO_5$ [M]$^+$ 359.0794, found: 359.0780.

3-(2-hydroxy-6-methoxybenzoyl)benzo[*g*]quinoline-5,10-dione (**3l**)

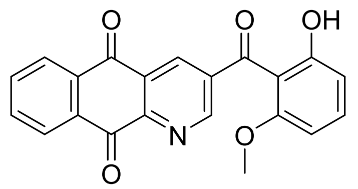

Brown solid (148 mg, 82%). ^{1}H NMR (500 MHz, TFA-d) δ 9.63 (s, 1H), 9.54 (s, 1H), 8.56 (d, J = 7.3 Hz, 2H), 8.19 – 8.09 (m, 2H), 7.76 – 7.67 (m, 1H), 6.89 (d, J = 8.4 Hz, 1H), 6.70 (d, J = 8.3 Hz, 1H), 3.68 (s, 3H). ^{13}C NMR (125 MHz, TFA-d) δ 193.46, 180.58, 177.39, 162.57, 148.24, 146.85, 146.57, 142.65, 142.54, 139.83, 138.92, 134.29, 133.38, 133.29, 130.96, 130.78, 115.89, 111.61, 105.59, 57.17. HRMS (EI$^+$): calcd for $C_{21}H_{13}NO_5$ [M]$^+$ 359.0794, found: 359.0786.

3-(2-hydroxy-5-methylbenzoyl)benzo[*g*]quinoline-5,10-dione (**3m**)

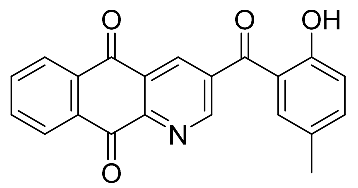

Brown solid (127 mg, 74%). ^{1}H NMR (400 MHz, TFA-d) δ 9.77 (s, 1H), 9.71 (s, 1H), 8.58 (d, J = 2.8 Hz, 1H), 8.57 (d, J = 2.5 Hz, 1H), 8.22 – 8.09 (m, 2H), 7.66 (d, J = 8.6 Hz, 1H), 7.35 (s, 1H), 7.21 (dd, J = 8.6, 2.0 Hz, 1H), 2.35 (s, 3H). ^{13}C NMR (125 MHz, TFA-d) δ 194.98, 180.42, 177.36, 161.82, 149.24, 147.73, 143.45, 143.27, 139.91, 139.00, 134.41, 134.30, 133.68, 133.62, 133.33, 131.01, 130.86, 120.78, 119.86, 20.74. HRMS (EI$^+$): calcd for $C_{21}H_{13}NO_4$ [M]$^+$ 343.0845, found: 343.0838.

3-(2-hydroxy-4,5-dimethylbenzoyl)benzo[*g*]quinoline-5,10-dione (**3n**)

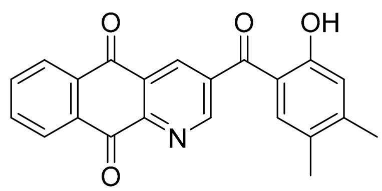

Brown solid (97 mg, 54%). ^{1}H NMR (400 MHz, TFA-d) δ 9.77 (d, J = 2.3 Hz, 1H), 9.70 (d, J = 2.3 Hz, 1H), 8.58 (dt, J = 7.3, 2.2 Hz, 2H), 8.22 – 8.11 (m, 2H), 7.28 (s, 1H), 7.12 (s, 1H),

2.45 (d, J = 2.8 Hz, 3H), 2.27 (d, J = 2.7 Hz, 3H). ^{13}C NMR (125 MHz, TFA-d) δ 194.28, 180.43, 177.35, 162.50, 155.37, 149.11, 147.70, 143.60, 143.37, 139.90, 138.99, 134.30, 134.07, 133.67, 133.61, 133.32, 131.00, 130.85, 121.44, 118.02, 21.46, 19.28. HRMS (EI$^+$): calcd for $C_{22}H_{15}NO_4$ [M]$^+$ 357.1001, found: 357.1001.

3-(5-chloro-2-hydroxy-4-methylbenzoyl)benzo[*g*]quinoline-5,10-dione (**3o**)

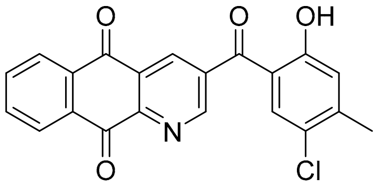

Brown solid (142 mg, 75%). ^{1}H NMR (400 MHz, TFA-d) δ 9.78 (d, J = 1.9 Hz, 1H), 9.71 (d, J = 2.0 Hz, 1H), 8.60 – 8.54 (m, 2H), 8.21 – 8.11 (m, 2H), 7.52 (s, 1H), 7.20 (s, 1H), 2.53 (s, 3H). ^{13}C NMR (125 MHz, TFA-d) δ 193.58, 180.45, 177.38, 162.69, 153.15, 149.28, 147.63, 143.59, 143.10, 139.92, 139.02, 134.29, 133.71, 133.42, 133.33, 131.02, 130.87, 129.47, 122.92, 119.03, 21.72. HRMS (EI$^+$): calcd for $C_{21}H_{12}ClNO_4$ [M]$^+$ 377.0455, found: 377.0447.

3-(2-hydroxy-4,5-dimethoxybenzoyl)benzo[*g*]quinoline-5,10-dione (**3p**)

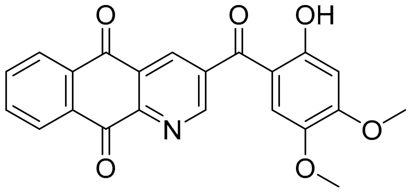

Brown solid (78 mg, 40%). ^{1}H NMR (600 MHz, TFA-d) δ 9.76 (s, 1H), 9.72 (s, 1H), 8.61 – 8.54 (m, 2H), 8.17 (t, J = 7.4 Hz, 1H), 8.14 (t, J = 7.4 Hz, 1H), 7.10 (s, 1H), 6.88 (s, 1H), 4.11 (s, 3H), 3.90 (s, 3H). ^{13}C NMR (125 MHz, TFA-d) δ 192.10, 180.42, 177.39, 164.78, 162.56, 149.26, 147.37, 144.83, 143.67, 143.35, 139.87, 138.97, 134.27, 133.65, 133.34, 130.99, 130.84, 116.51, 112.44, 103.41, 59.17, 57.98. HRMS (EI$^+$): calcd for $C_{22}H_{15}NO_6$ [M]$^+$ 389.0899, found: 389.0891.

3-(2-hydroxy-5-morpholinobenzoyl)benzo[*g*]quinoline-5,10-dione (**3q**)

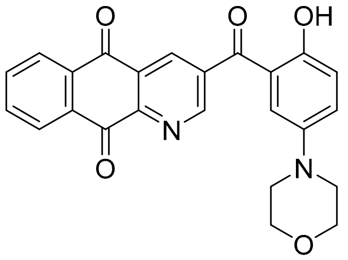

Brown solid (142 mg, 69%). ^{1}H NMR (400 MHz, TFA-d) δ 9.81 (s, 1H), 9.76 (s, 1H), 8.59 (d, J = 6.5 Hz, 2H), 8.22 – 8.12 (m, 3H), 8.05 (d, J = 9.4 Hz, 1H), 7.51 (d, J = 9.3 Hz, 1H), 4.42 (s, 4H), 4.00 (s, 4H). ^{13}C NMR (125 MHz, TFA-d) δ 192.44, 180.52, 177.44, 149.90, 147.56, 143.92, 142.24, 139.97, 139.06, 134.91, 134.26, 133.64, 133.33, 131.33, 131.14, 130.89, 127.20, 124.13, 121.87, 66.56, 58.28. HRMS (EI$^+$): calcd for $C_{24}H_{18}N_2O_5$ [M]$^+$ 414.1216, found: 414.1212.

3-(2-hydroxy-1-naphthoyl)benzo[*g*]quinoline-5,10-dione (**3r**)

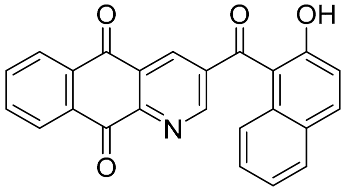

Brown solid (181 mg, 95%). ^{1}H NMR (600 MHz, TFA-d) δ 9.75 (d, *J* = 1.8 Hz, 1H), 9.62 (d, *J* = 1.8 Hz, 1H), 8.53 (dd, *J* = 7.4, 1.6 Hz, 1H), 8.51 (dd, *J* = 7.4, 1.6 Hz, 1H), 8.16 – 8.09 (m, 3H), 7.90 (dd, *J* = 7.1, 2.2 Hz, 1H), 7.67 (d, *J* = 8.7 Hz, 1H), 7.46 (tt, *J* = 7.1, 5.4 Hz, 2H), 7.27 (d, *J* = 9.0 Hz, 1H). ^{13}C NMR (150 MHz, TFA-d) δ 193.30, 180.41, 177.33, 161.62, 149.80, 148.43, 144.72, 143.24, 141.23, 139.88, 138.92, 134.23, 133.77, 133.41, 133.25, 131.67, 131.59, 131.52, 130.97, 130.78, 127.67, 125.92, 119.75, 115.50. HRMS (EI$^+$): calcd for $C_{24}H_{13}NO_4$ $[M]^+$ 379.0845, found: 379.0812.

3-(1-hydroxy-2-naphthoyl)benzo[*g*]quinoline-5,10-dione (**3s**)

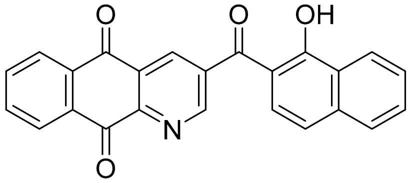

Brown solid (175 mg, 92%). ^{1}H NMR (400 MHz, TFA-d) δ 9.81 (s, 1H), 9.74 (s, 1H), 8.57 (t, *J* = 6.6 Hz, 3H), 8.21 – 8.09 (m, 2H), 7.83 (d, *J* = 6.3 Hz, 2H), 7.68 (t, *J* = 7.2 Hz, 1H), 7.40 (d, *J* = 8.7 Hz, 1H), 7.30 (d, *J* = 8.5 Hz, 1H). ^{13}C NMR (125 MHz, TFA-d) δ 193.82, 180.41, 177.37, 168.46, 149.07, 147.72, 143.85, 143.23, 141.06, 139.90, 138.98, 134.96, 134.29, 133.60, 133.32, 131.00, 130.85, 129.93, 129.52, 126.89, 126.77, 125.74, 123.05, 113.45. HRMS (EI$^+$): calcd for $C_{24}H_{13}NO_4$ $[M]^+$ 379.0845, found: 379.0829.

3-(2-hydroxybenzoyl)-7,8-dimethoxybenzo[*g*]quinoline-5,10-dione (**3t**)

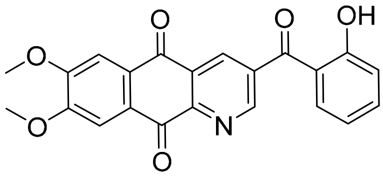

Brown solid (132 mg, 68%). ^{1}H NMR (500 MHz, TFA-d) δ 9.71 (s, 1H), 9.63 (s, 1H), 8.04 (s, 1H), 8.03 (s, 1H), 7.81 (t, *J* = 8.0 Hz, 1H), 7.58 (d, *J* = 8.0 Hz, 1H), 7.30 (d, *J* = 8.5 Hz, 1H), 7.18 (t, *J* = 7.7 Hz, 1H), 4.25 (d, *J* = 3.3 Hz, 6H). ^{13}C NMR (125 MHz, TFA-d) δ 195.04, 179.45, 175.96, 164.04, 158.82, 157.82, 148.49, 147.32, 143.89, 143.09, 141.92, 134.31, 133.64, 130.31, 128.93, 123.55, 121.00, 120.03, 112.03, 111.60, 58.36. HRMS (EI$^+$): calcd for $C_{22}H_{15}NO_6$ $[M]^+$ 389.0899, found: 389.0885.

VI. NMR spectra of Compounds

^{1}H NMR of compound **3a**

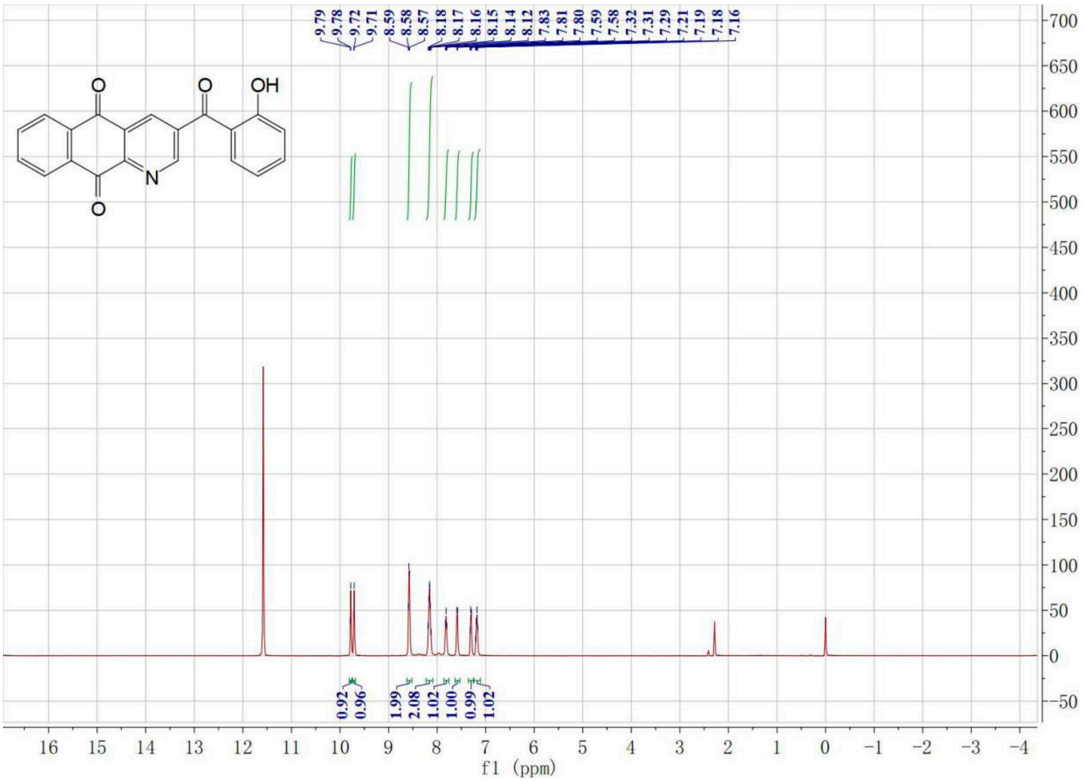

^{13}C NMR of compound **3a**

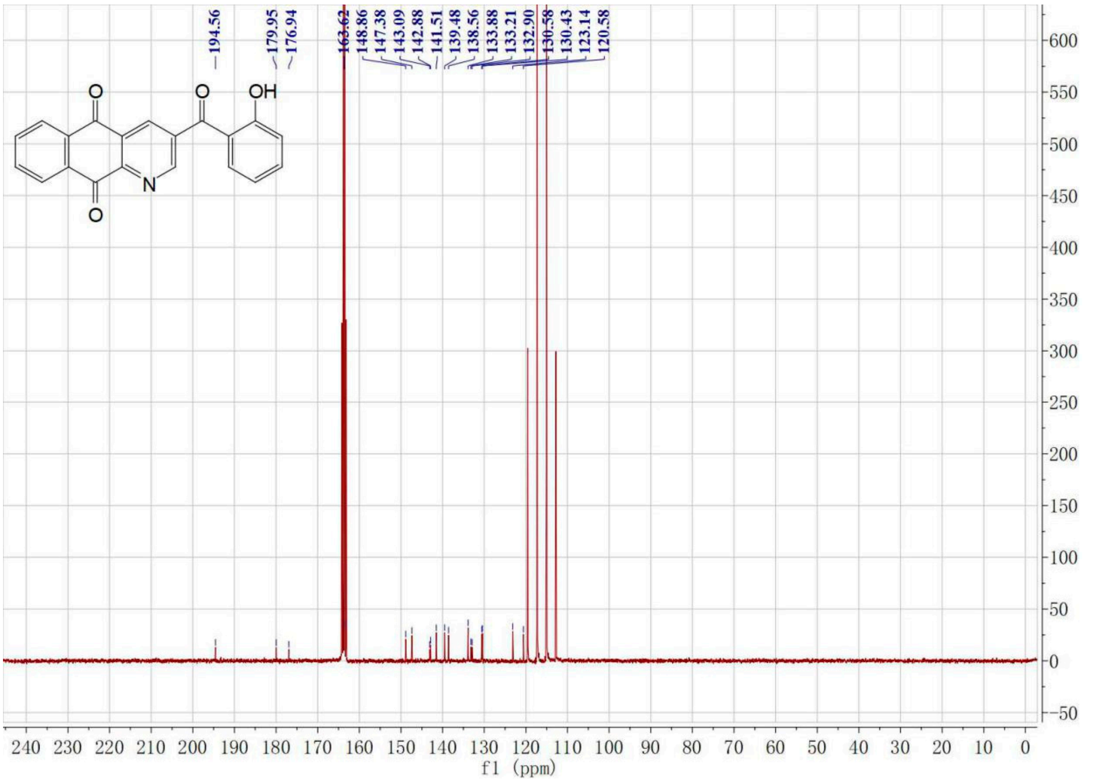

¹H NMR of compound **3b**

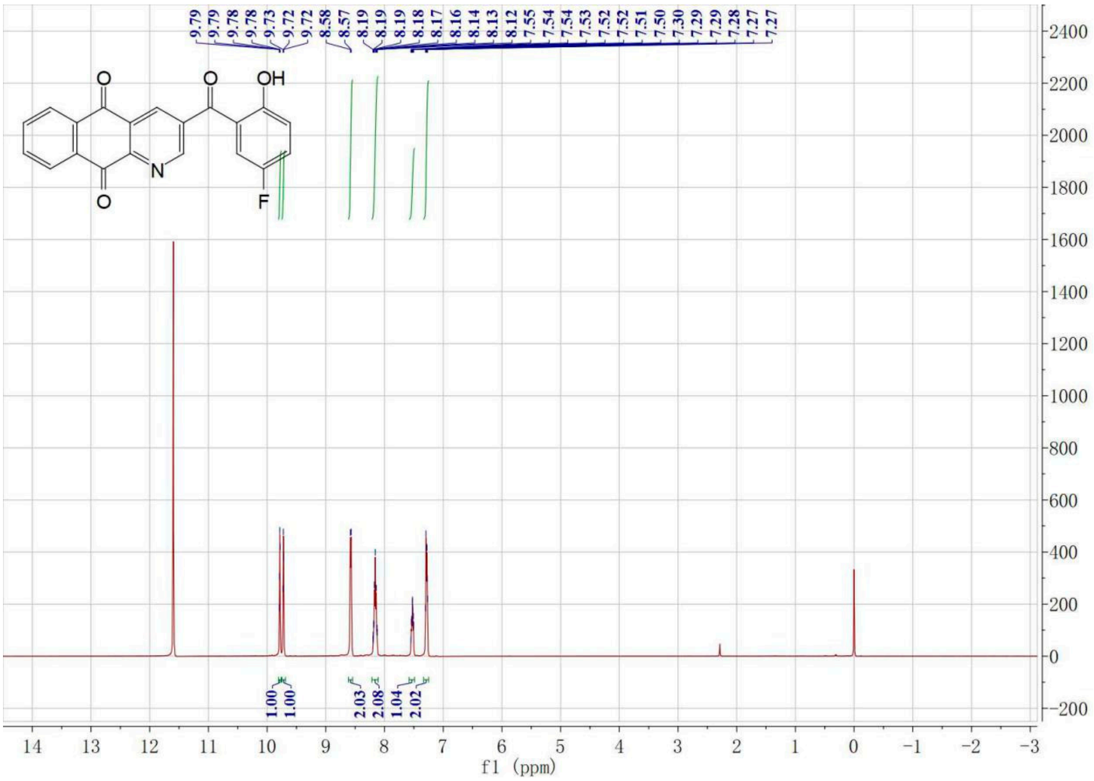

¹³C NMR of compound **3b**

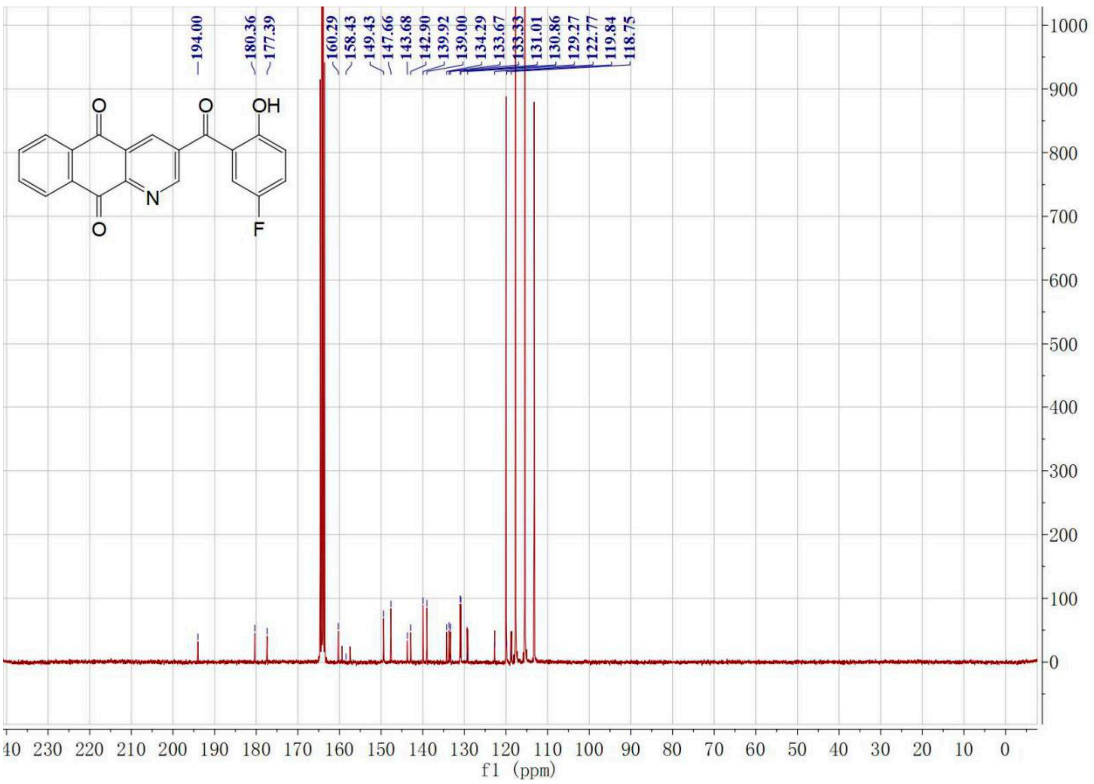

^{1}H NMR of compound **3c**

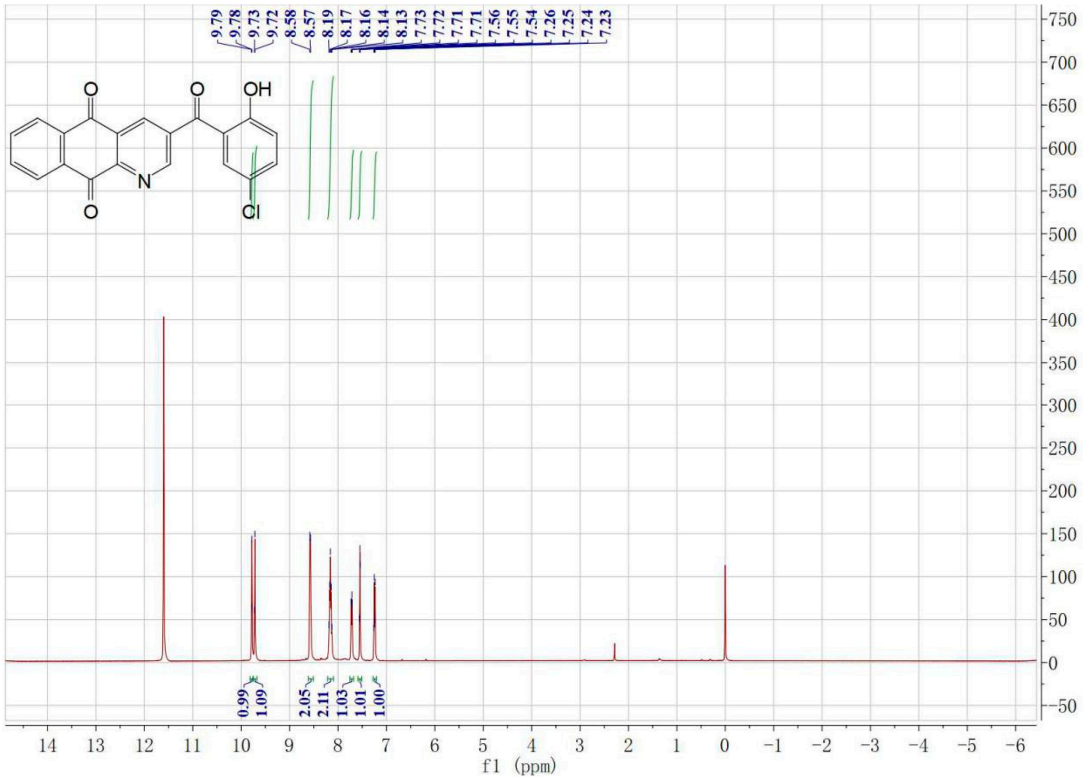

^{13}C NMR of compound **3c**

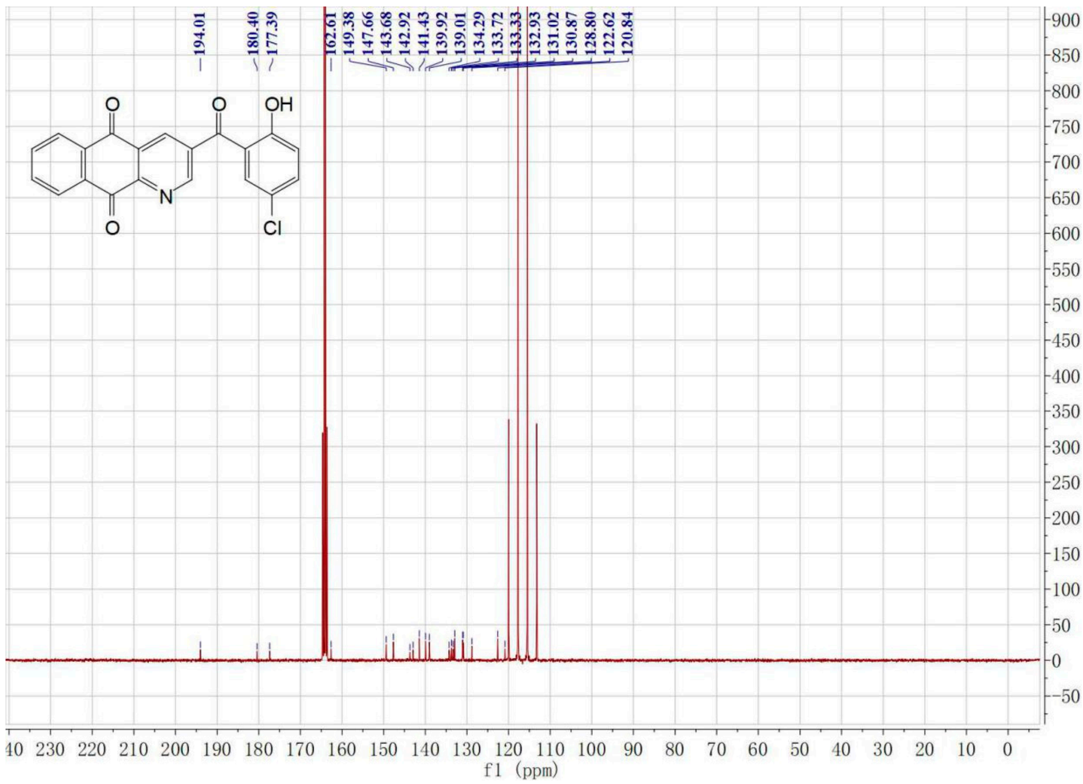

^{1}H NMR of compound **3d**

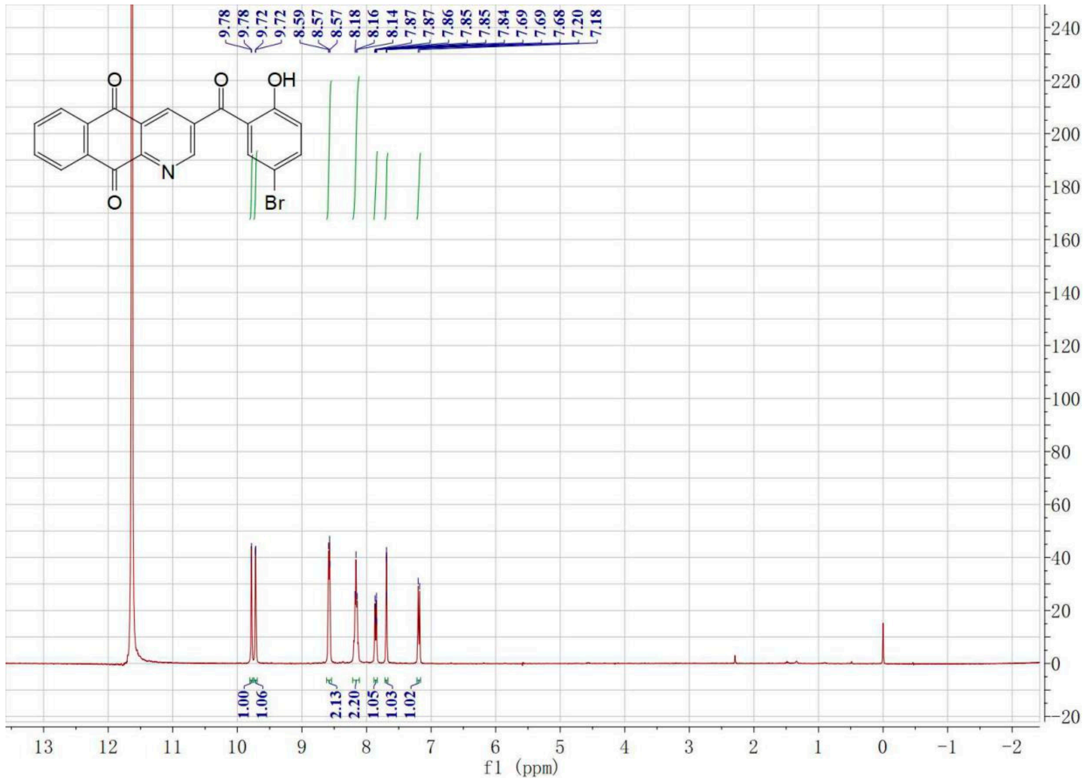

^{13}C NMR of compound **3d**

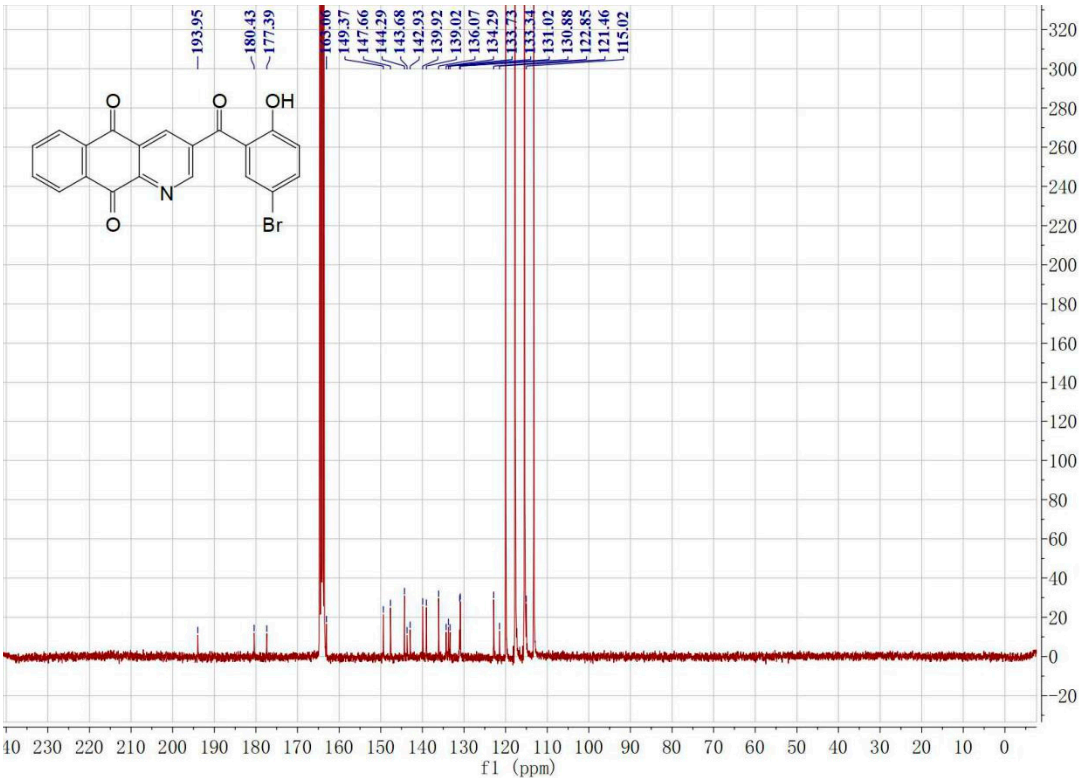

^{1}H NMR of compound **3e**

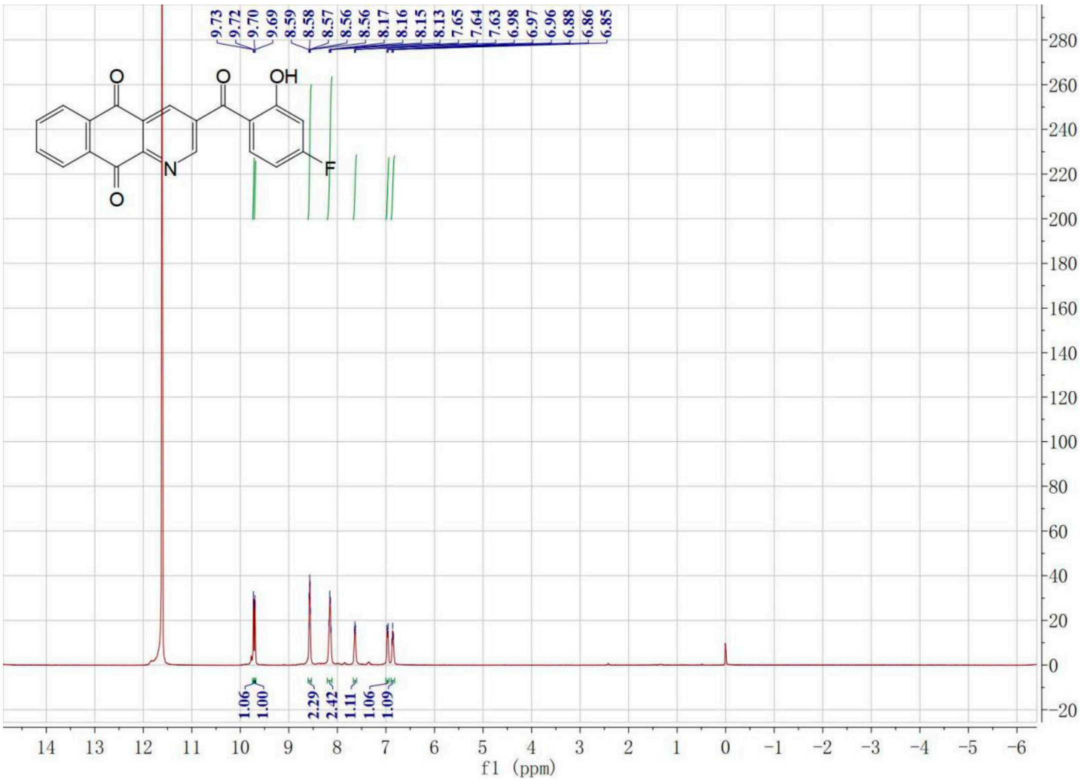

^{13}C NMR of compound **3e**

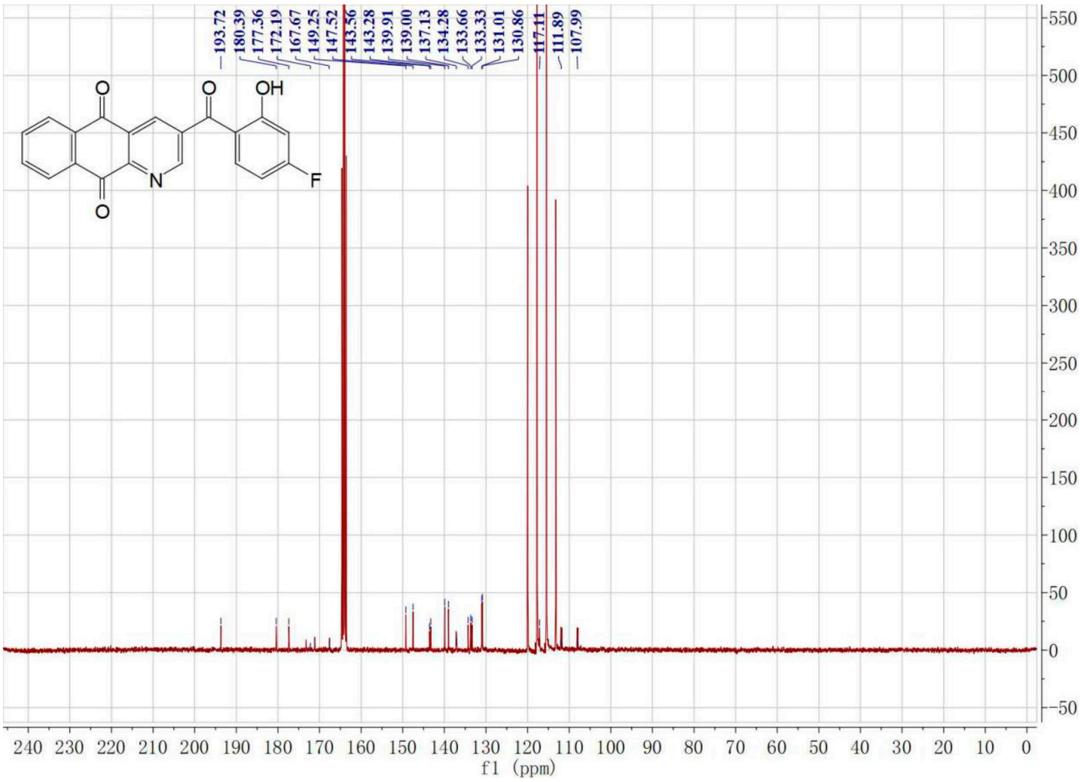

^{1}H NMR of compound **3f**

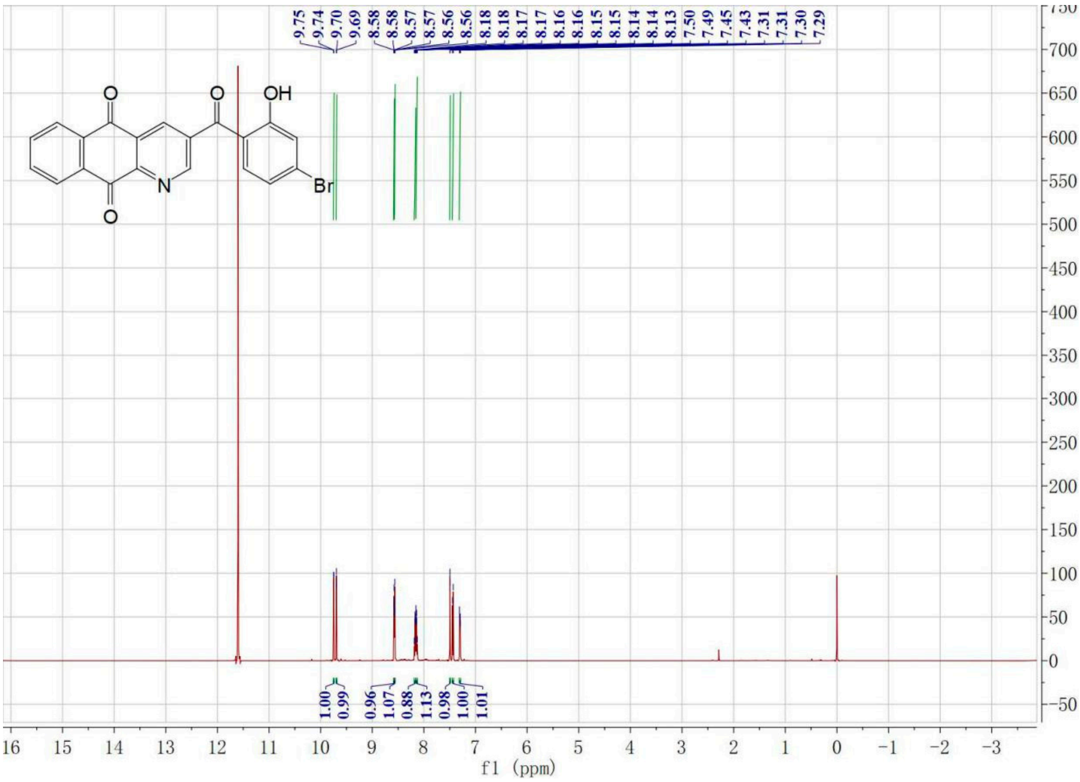

^{13}C NMR of compound **3f**

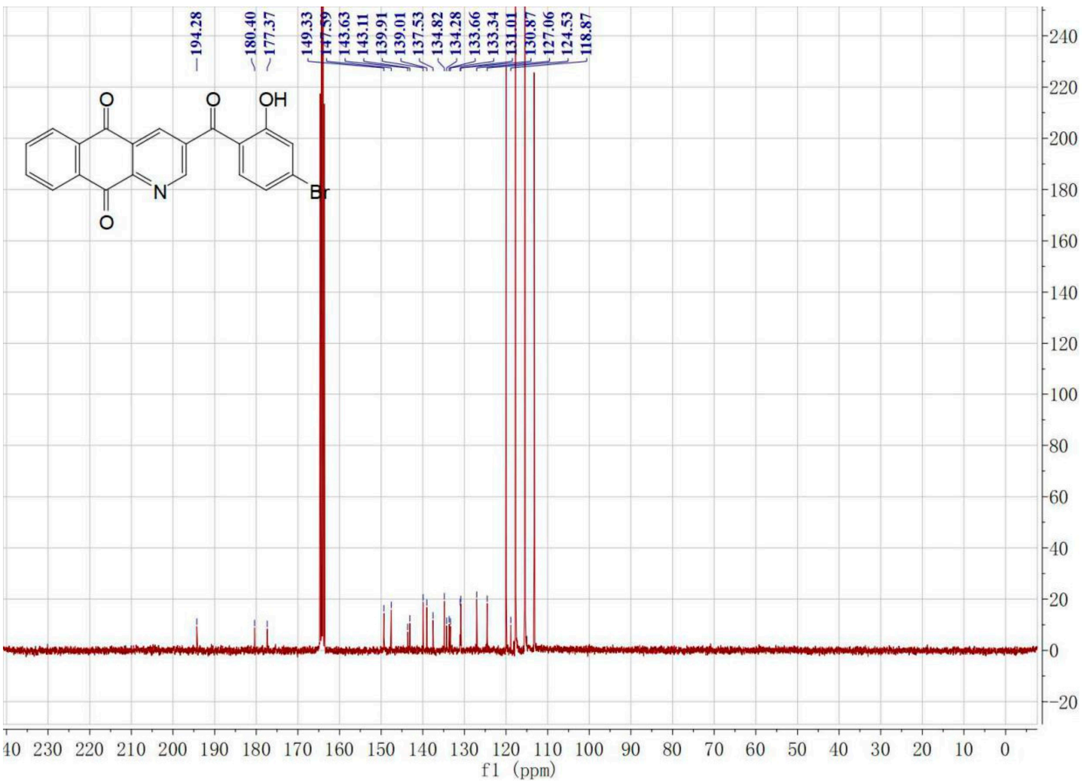

^{1}H NMR of compound **3g**

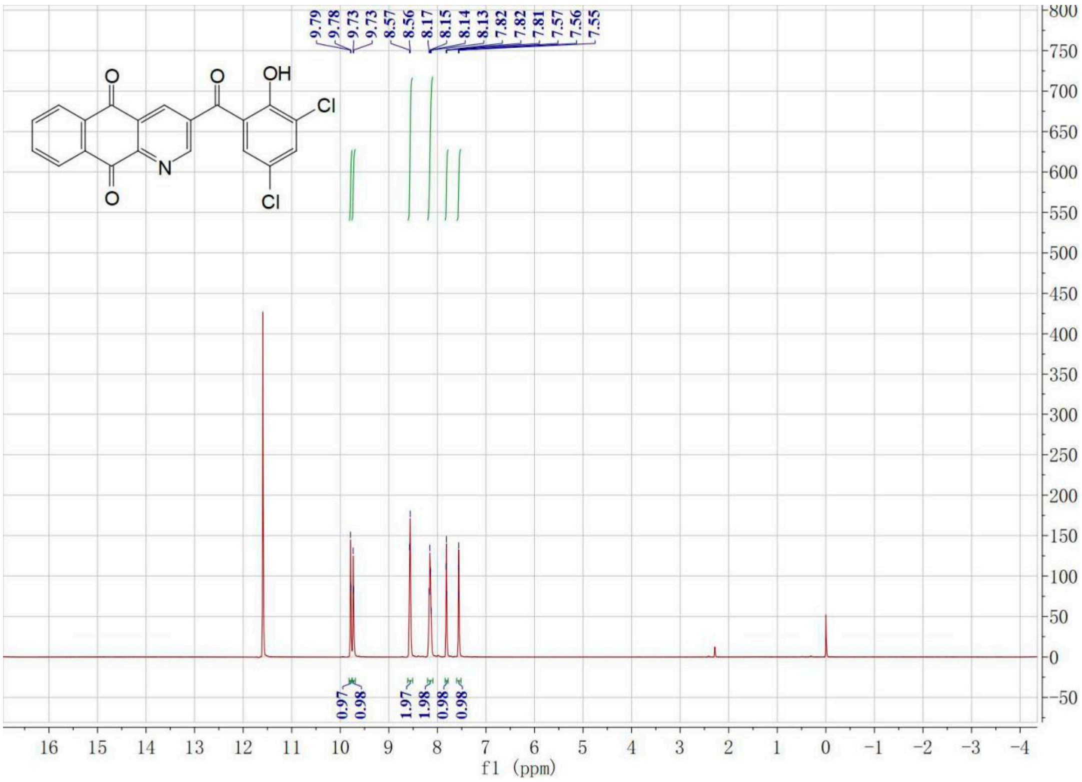

^{13}C NMR of compound **3g**

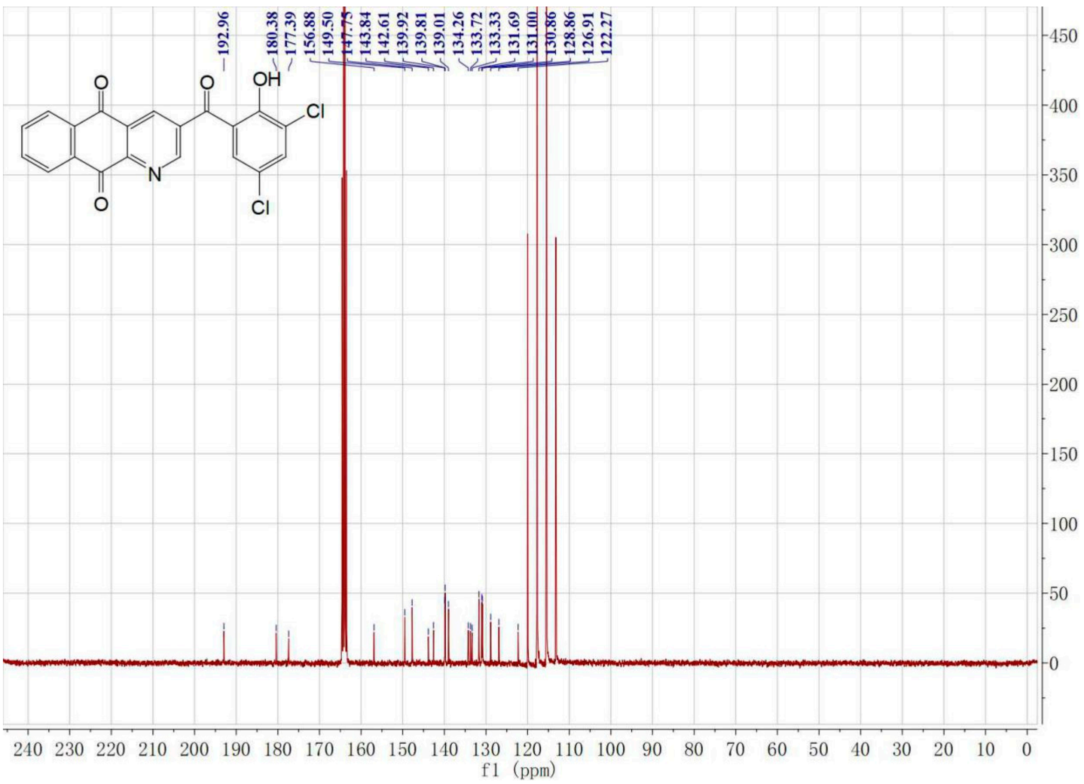

^{1}H NMR of compound **3h**

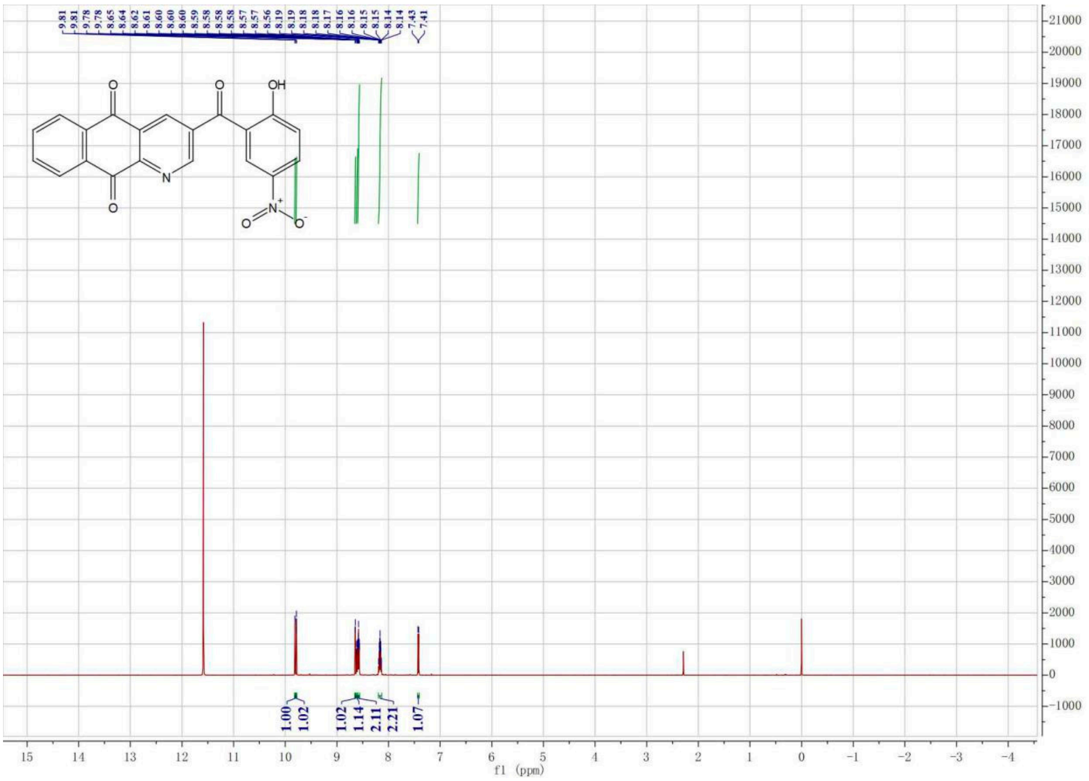

^{13}C NMR of compound **3h**

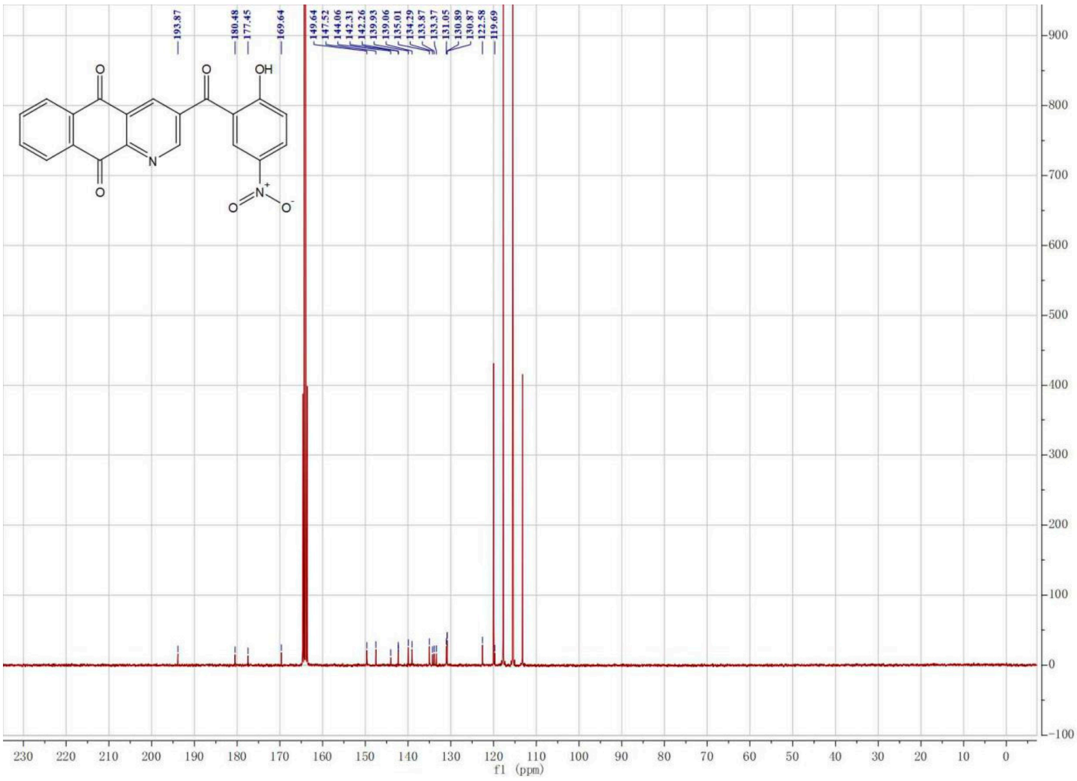

^{1}H NMR of compound **3i**

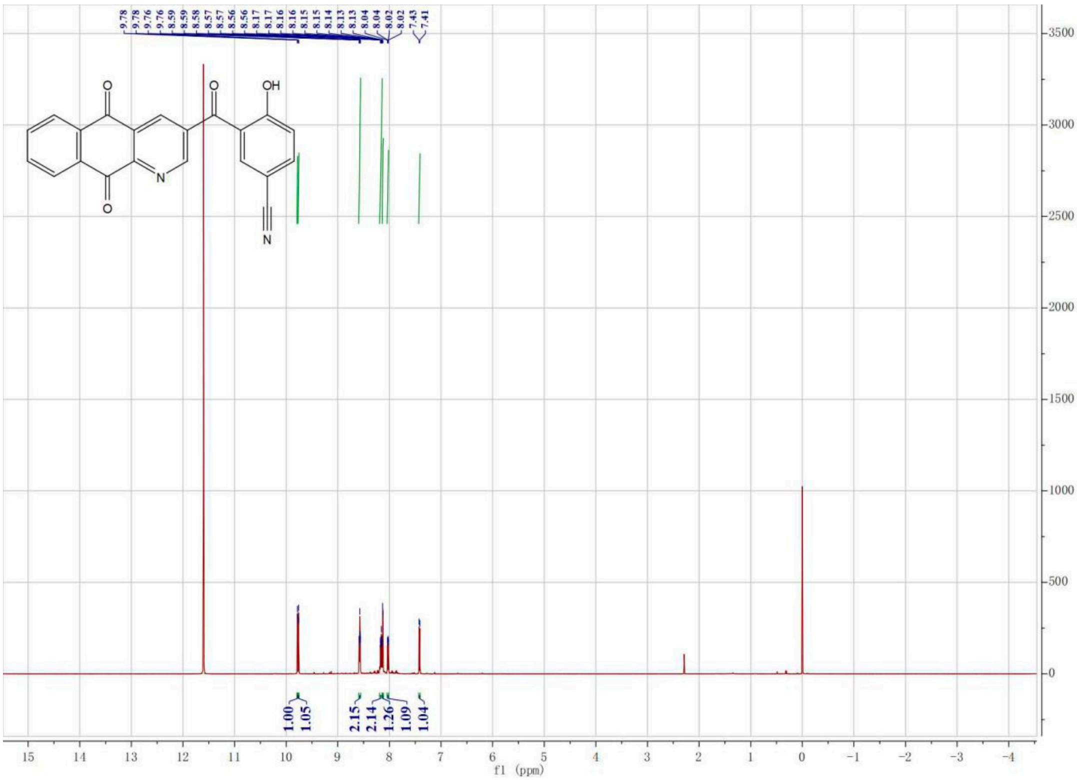

^{13}C NMR of compound **3i**

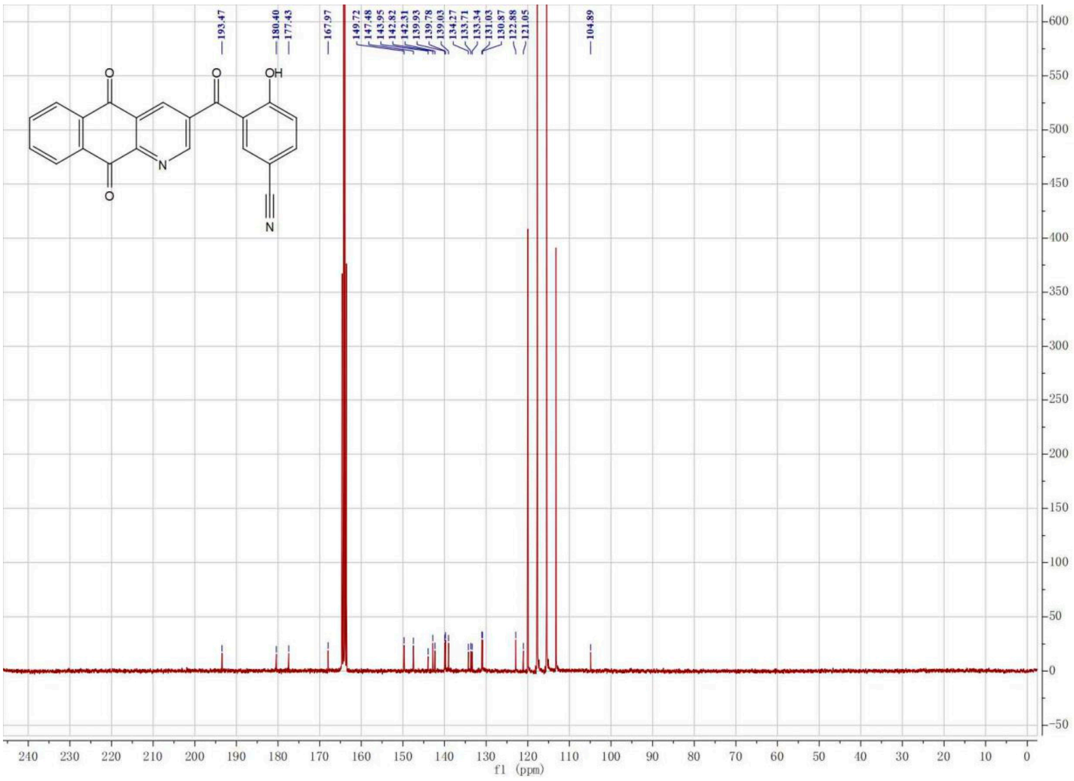

^{1}H NMR of compound **3j**

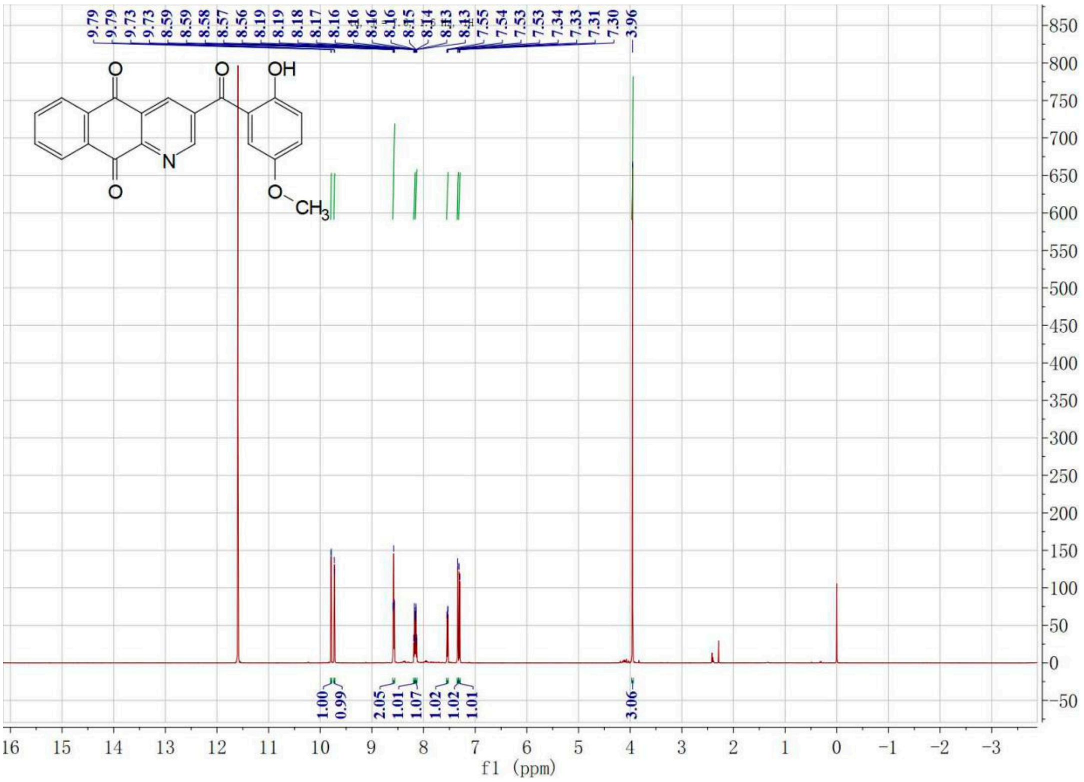

^{13}C NMR of compound **3j**

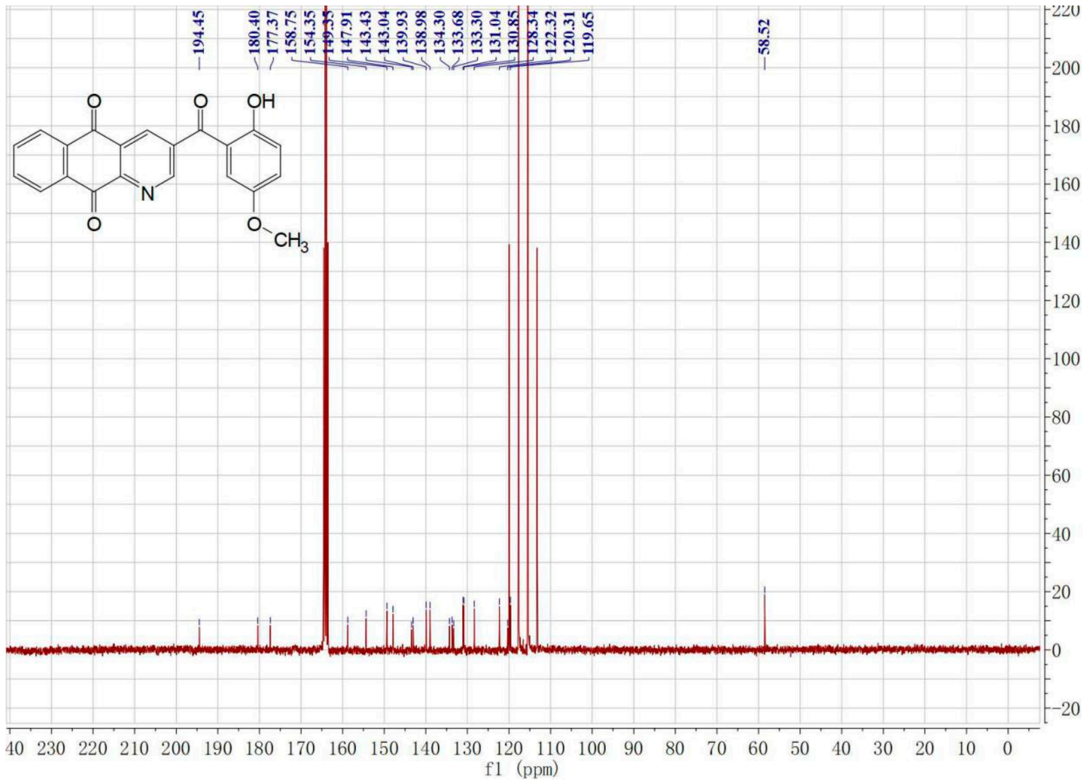

^{1}H NMR of compound **3k**

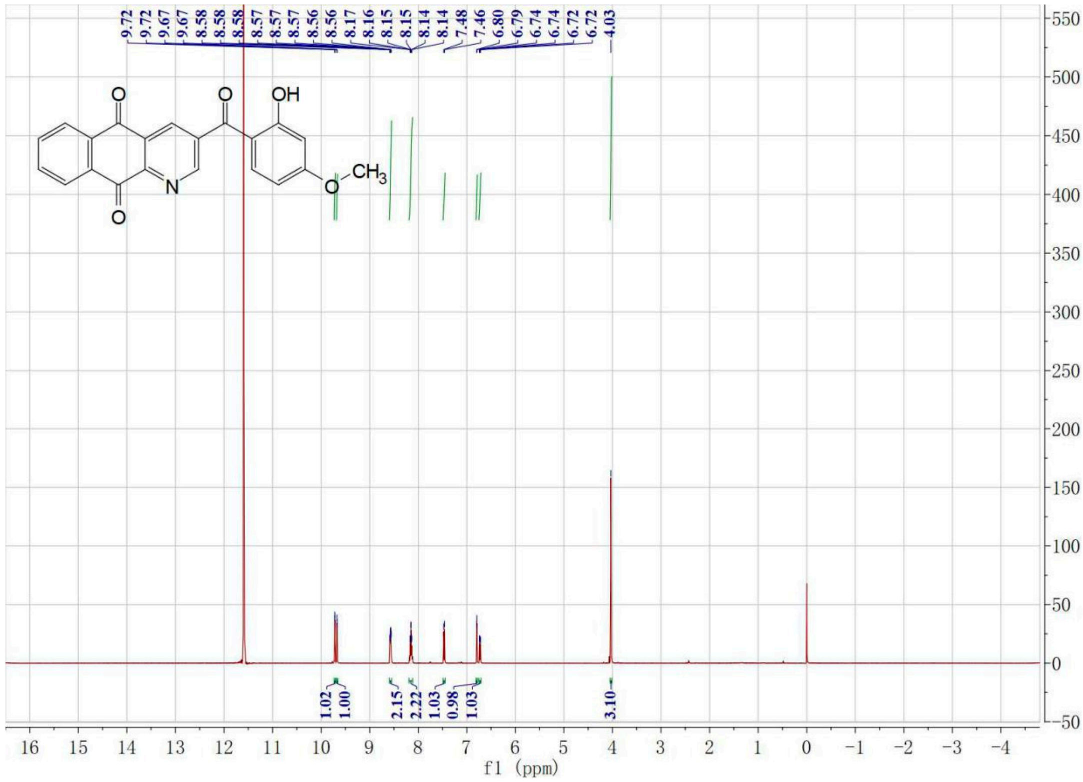

^{13}C NMR of compound **3k**

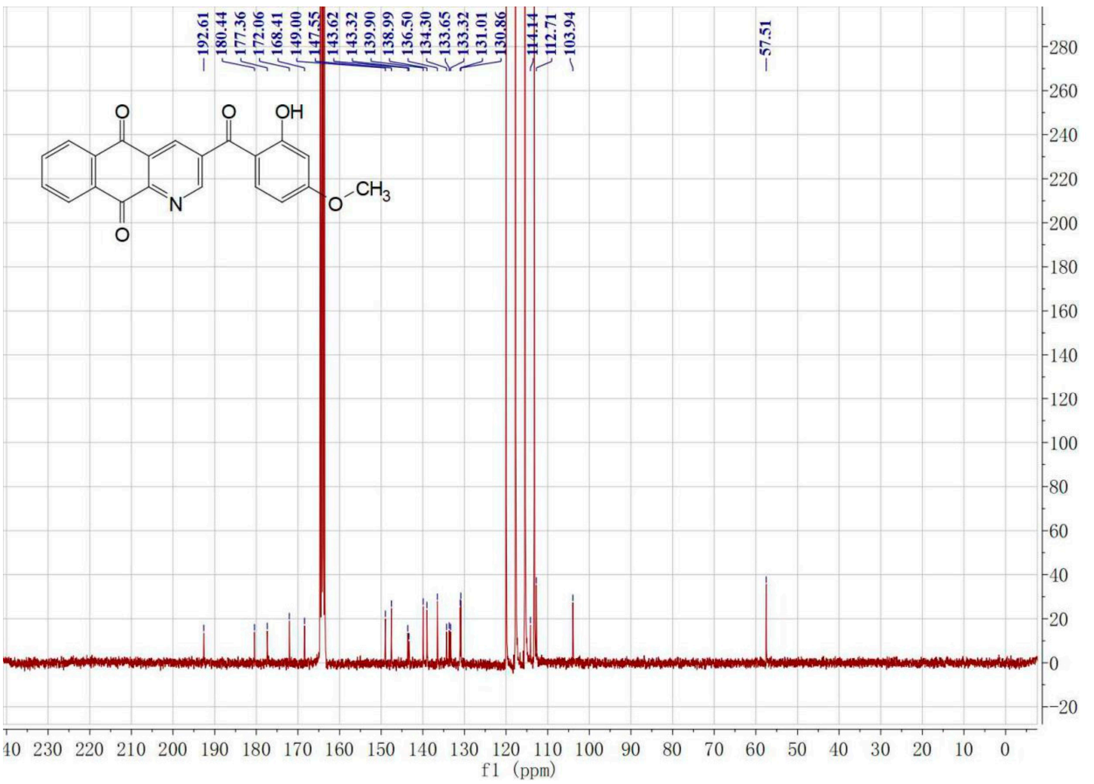

^{1}H NMR of compound **3l**

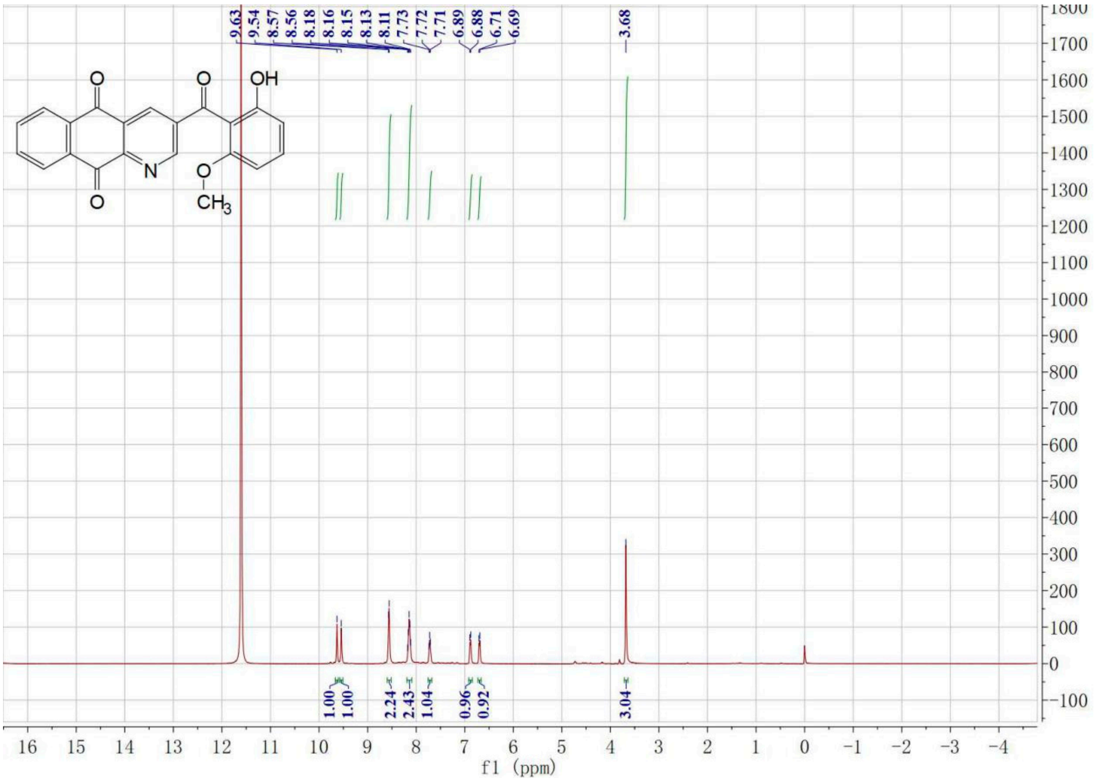

^{13}C NMR of compound **3l**

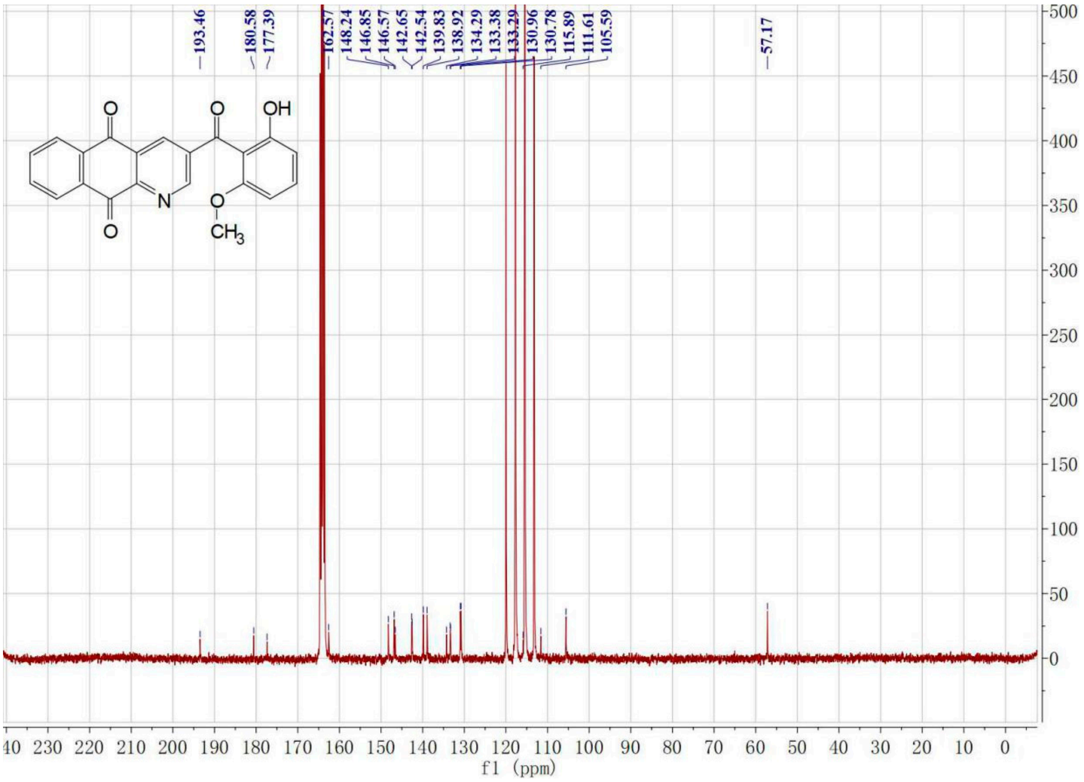

^{1}H NMR of compound **3m**

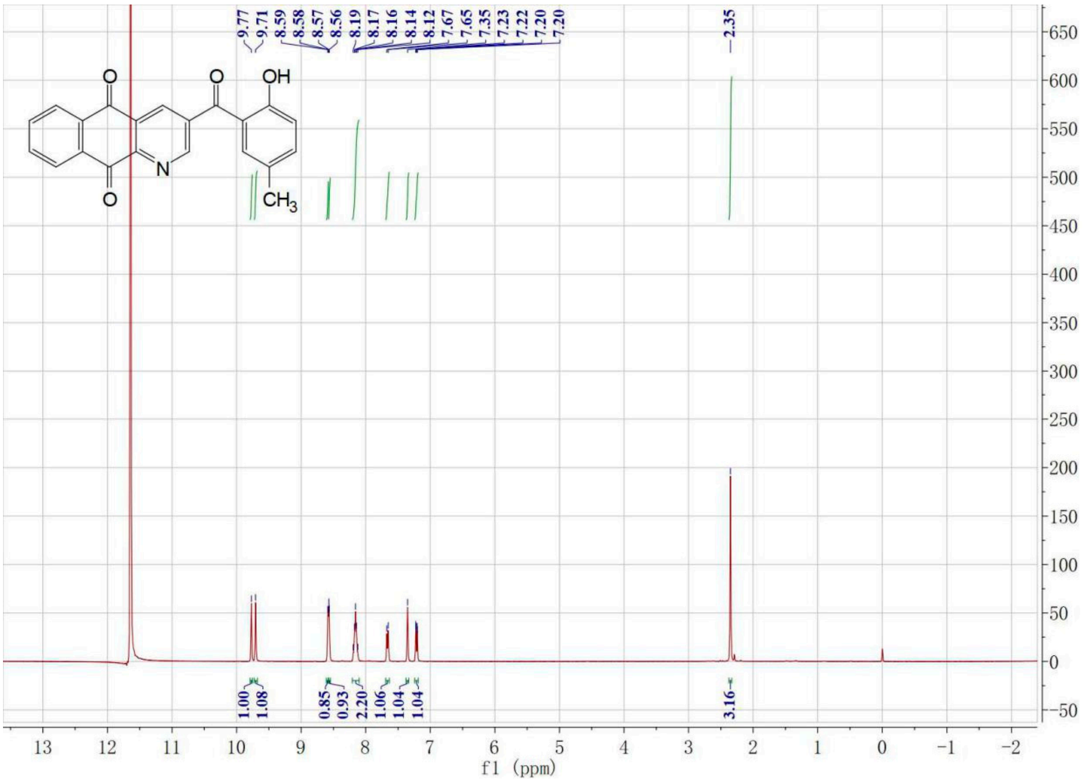

^{13}C NMR of compound **3m**

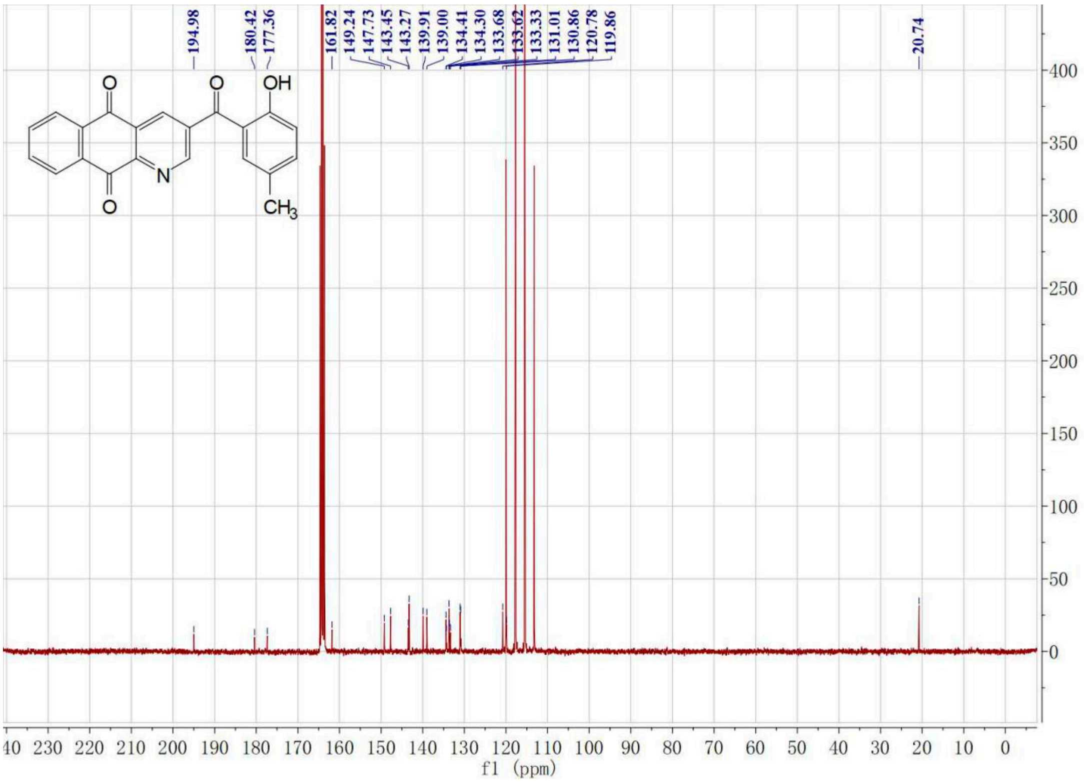

^{1}H NMR of compound **3n**

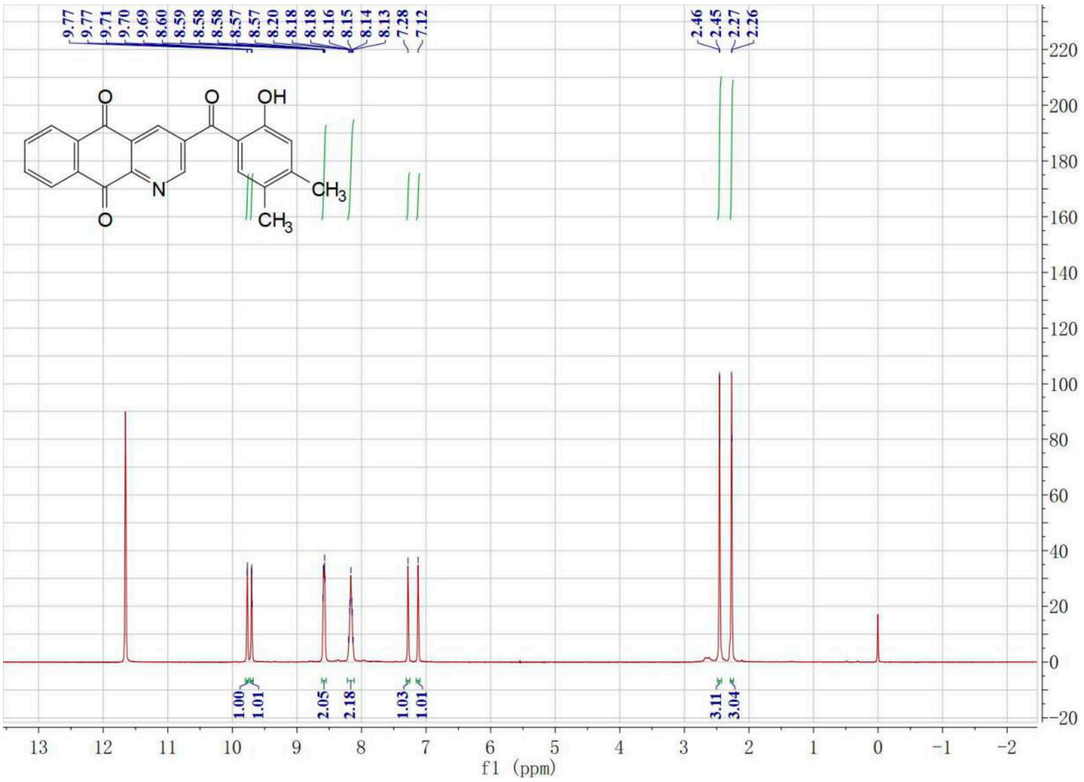

^{13}C NMR of compound **3n**

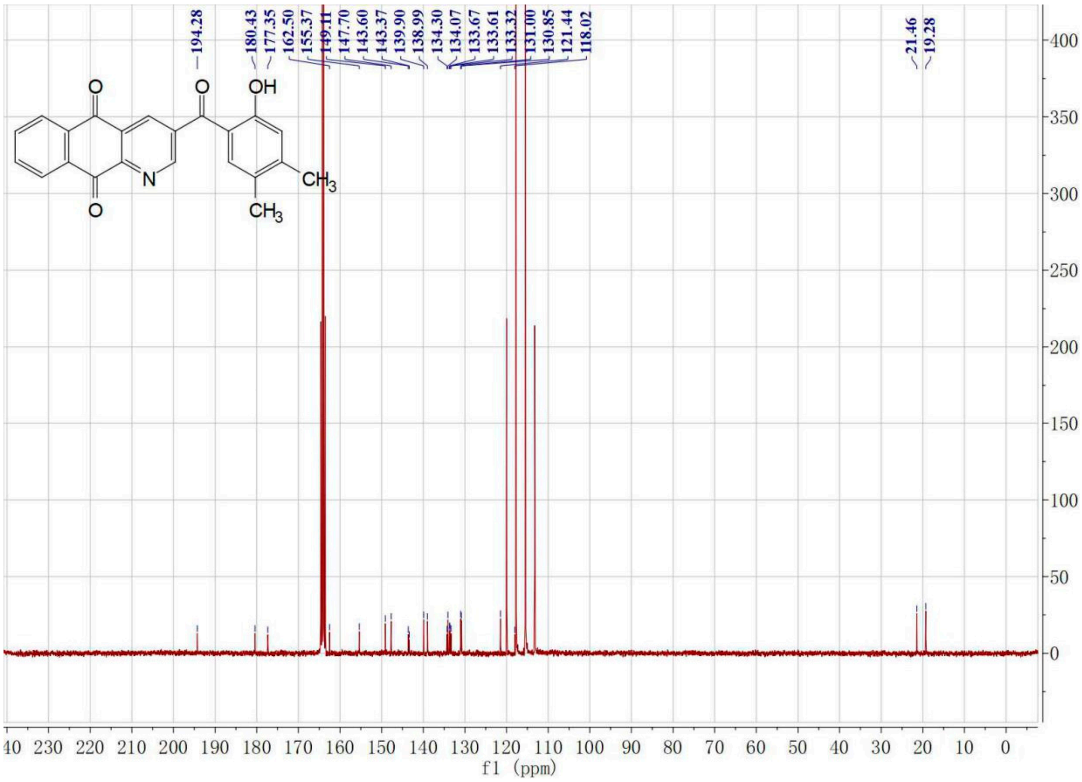

^{1}H NMR of compound **3o**

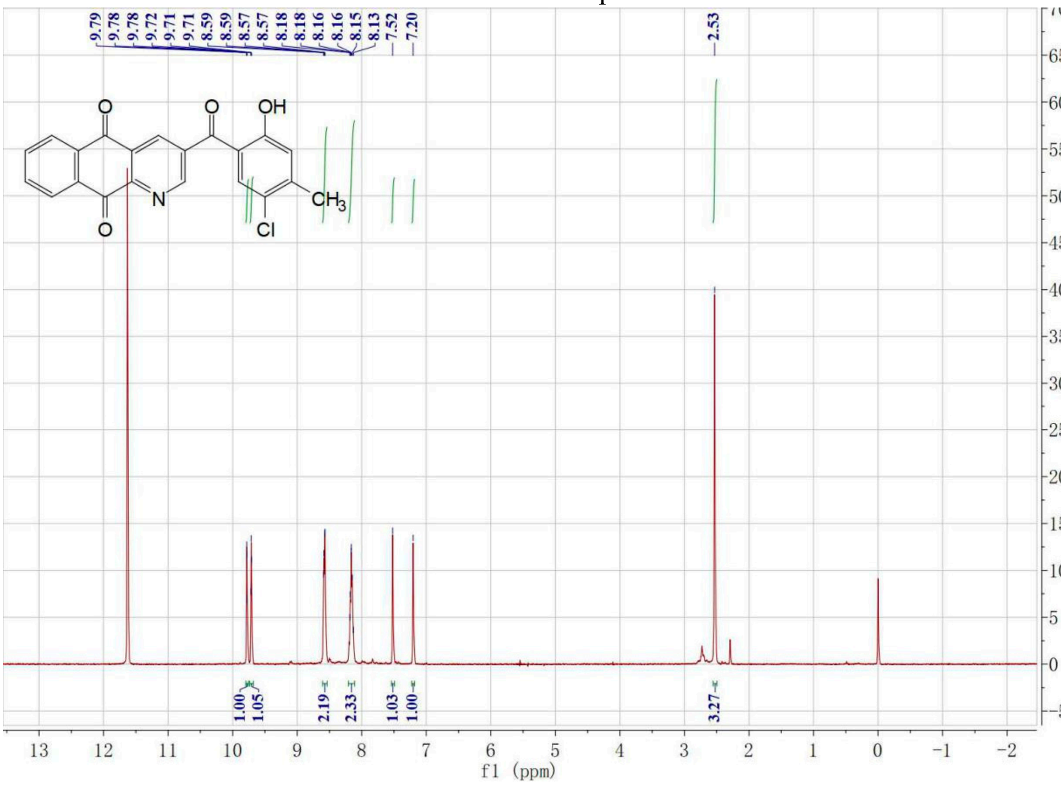

^{13}C NMR of compound **3o**

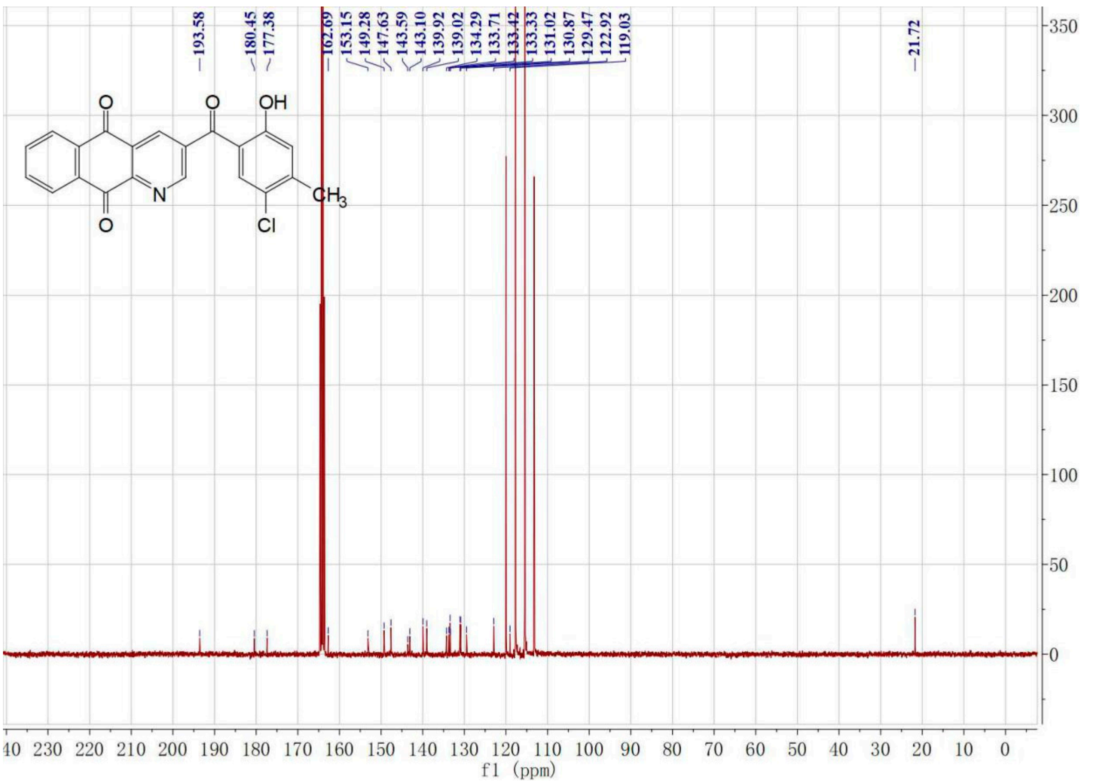

^{1}H NMR of compound **3p**

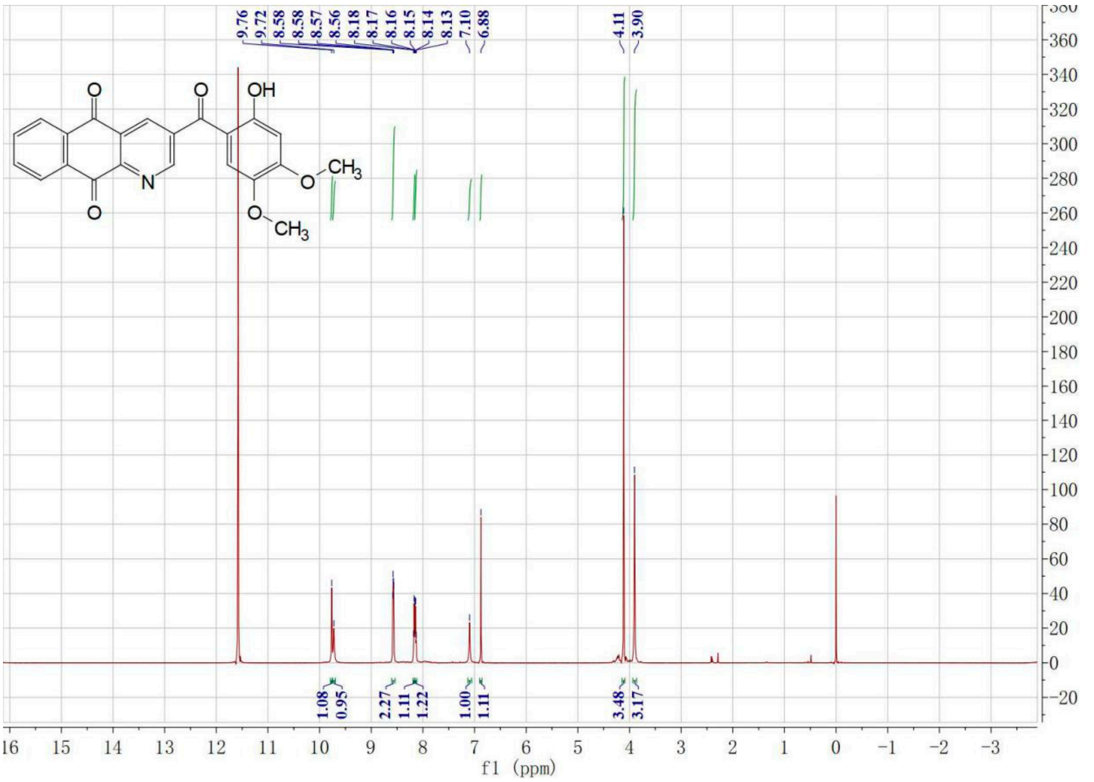

^{13}C NMR of compound **3p**

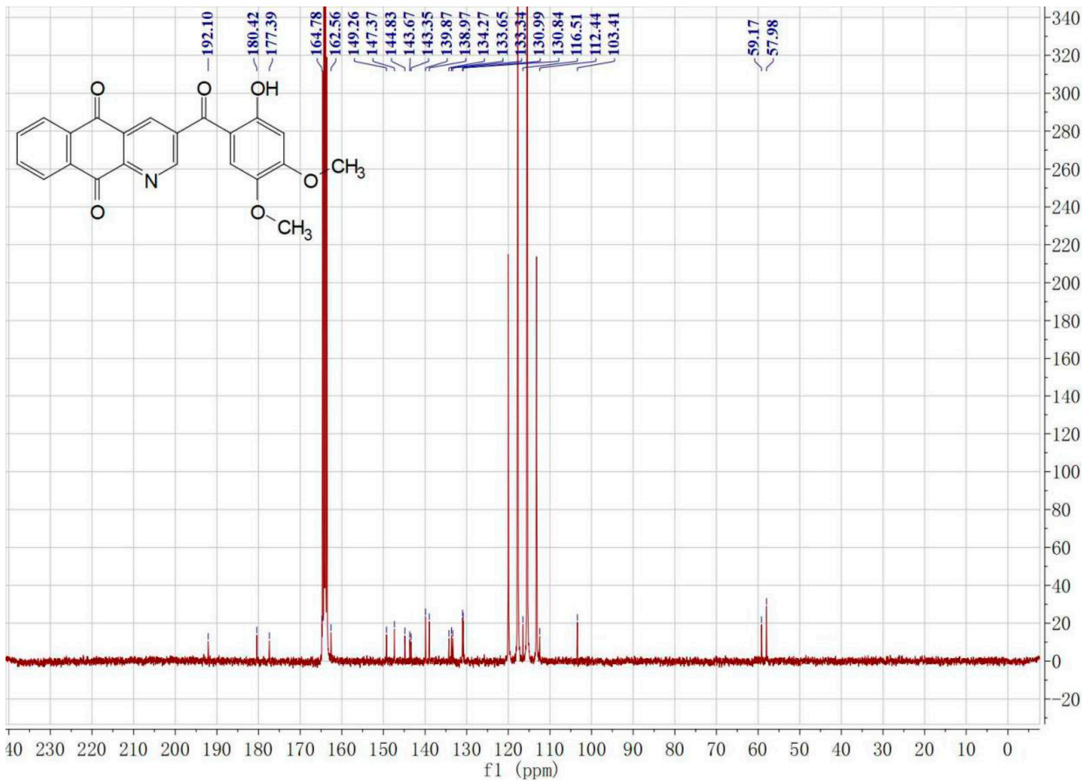

^{1}H NMR of compound **3q**

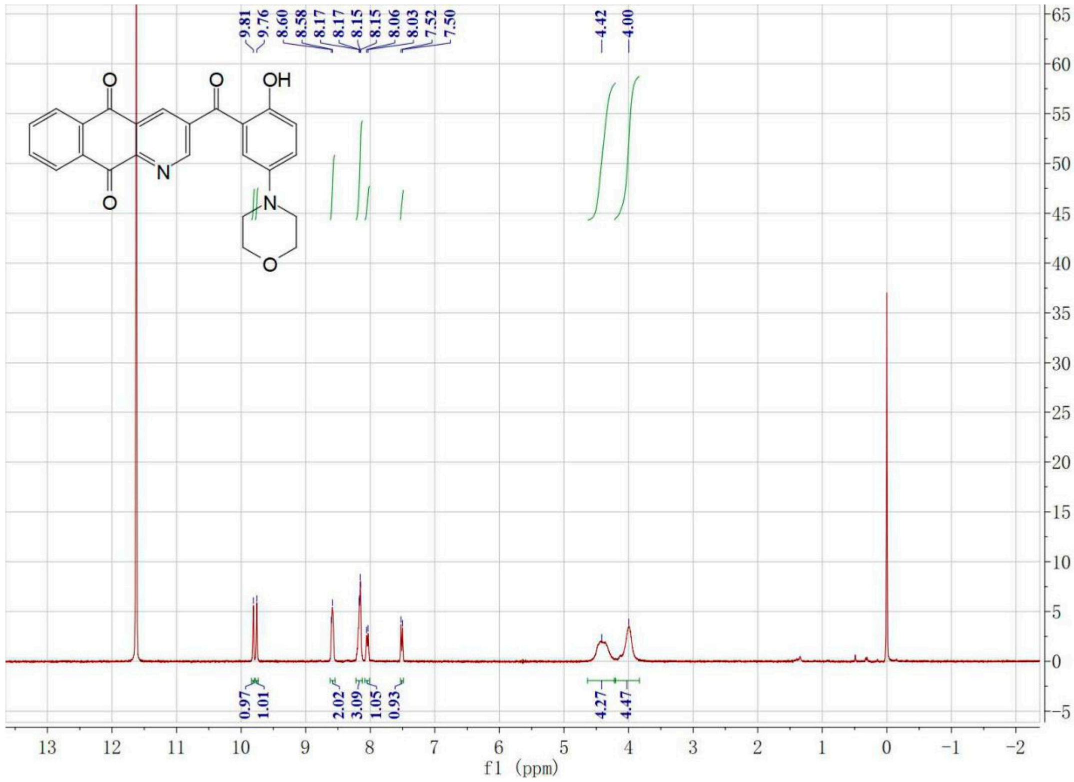

^{13}C NMR of compound **3q**

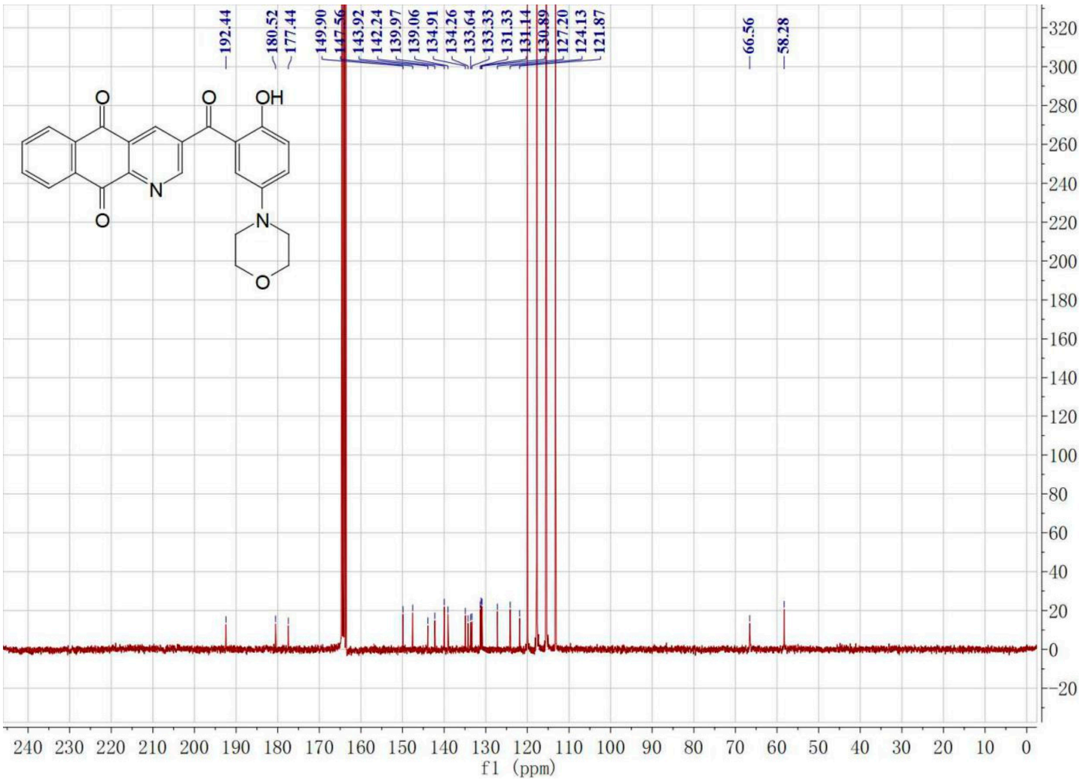

^{1}H NMR of compound **3r**

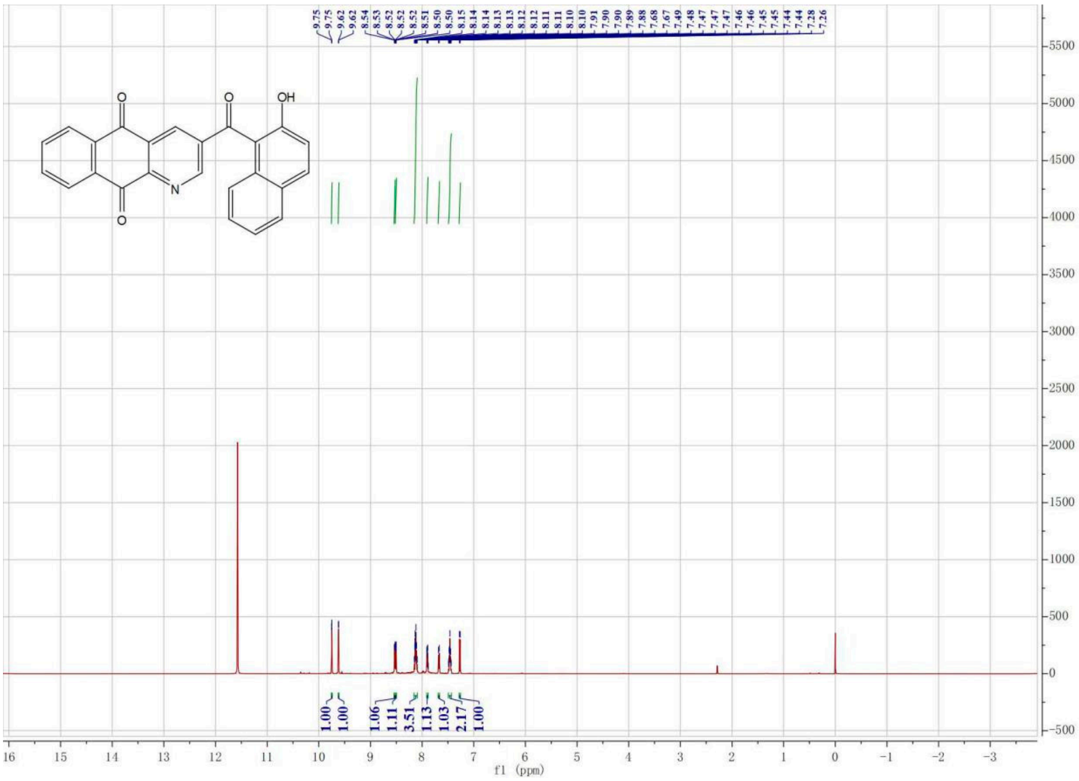

^{13}C NMR of compound **3r**

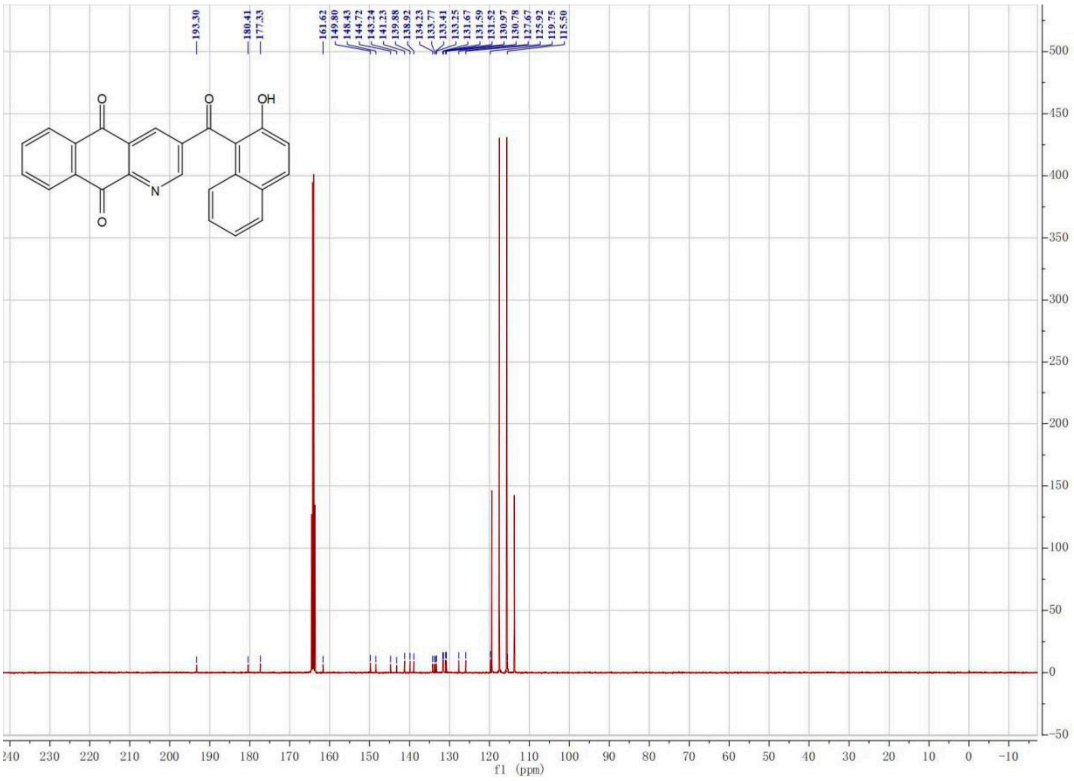

^{1}H NMR of compound **3s**

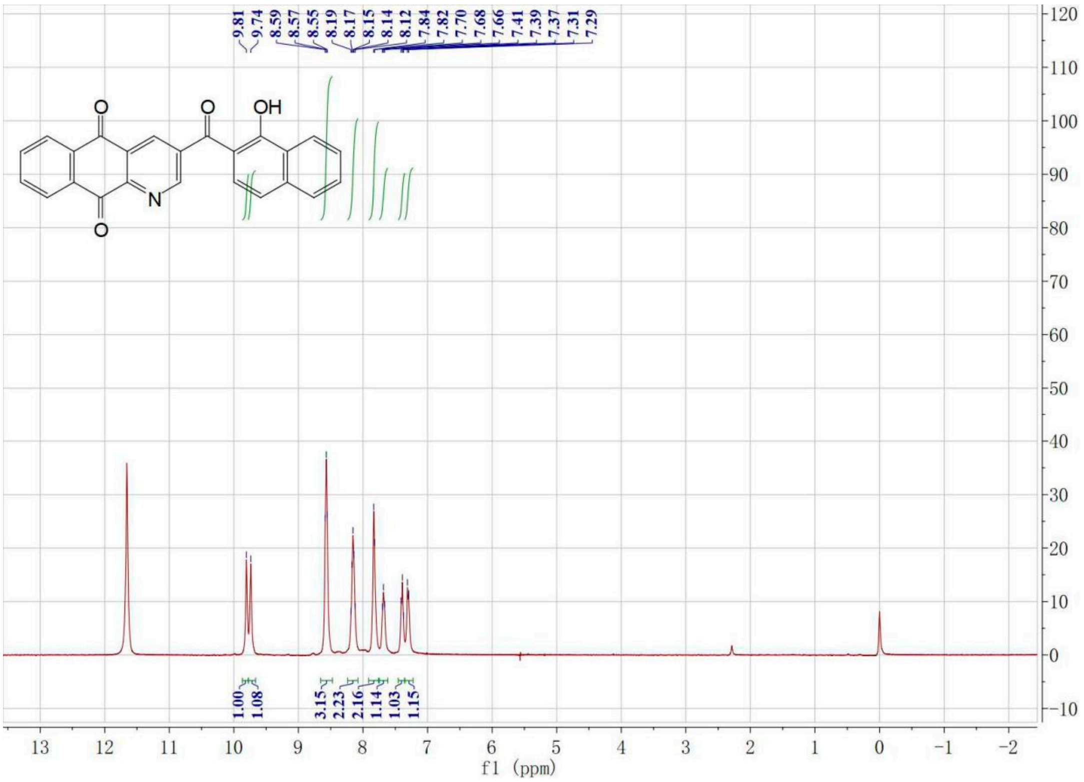

^{13}C NMR of compound **3s**

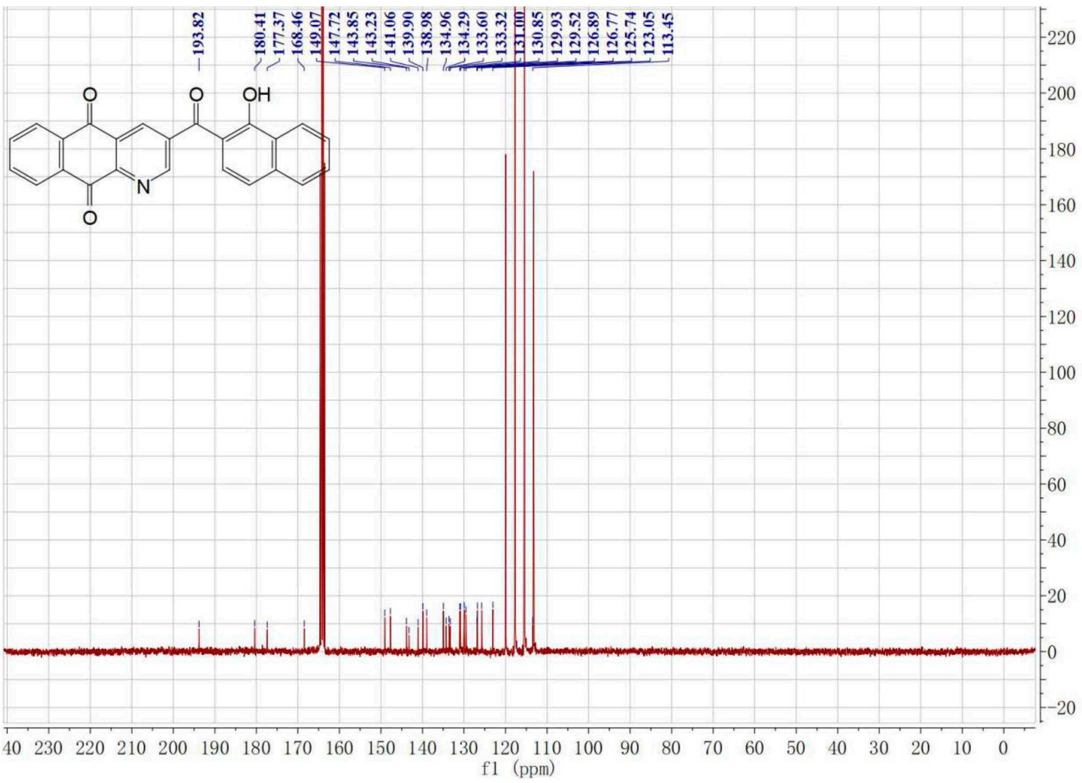

^{1}H NMR of compound **3t**

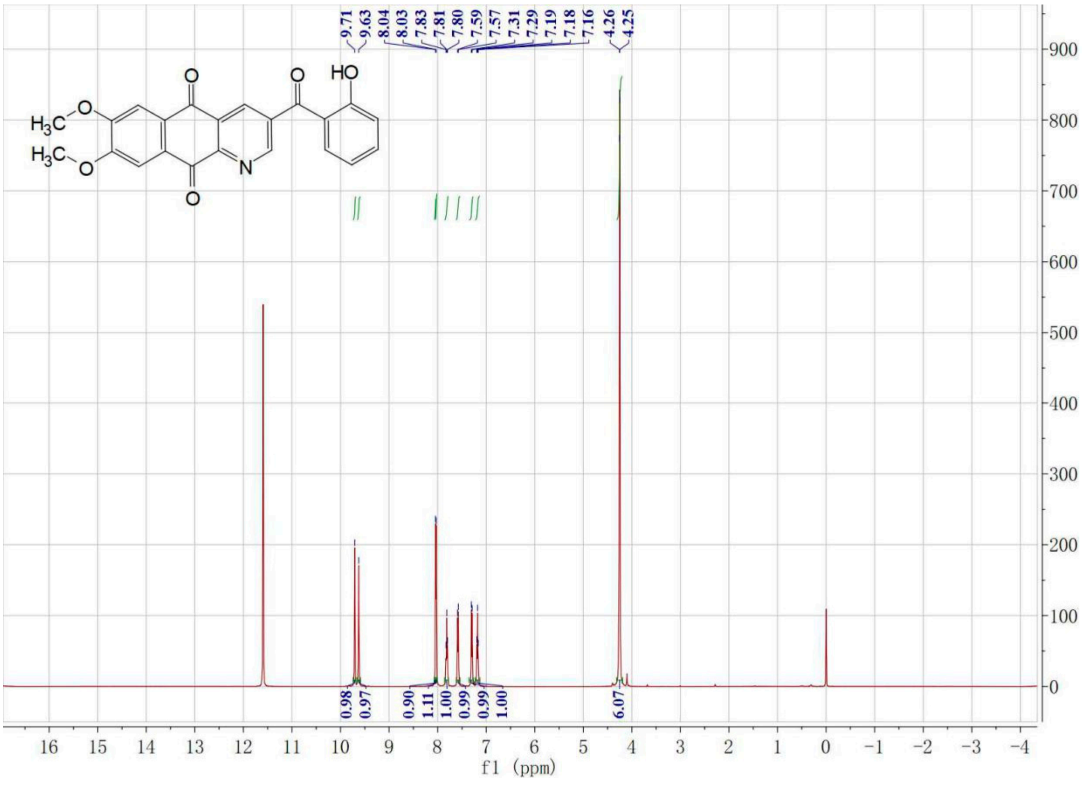

^{13}C NMR of compound **3t**

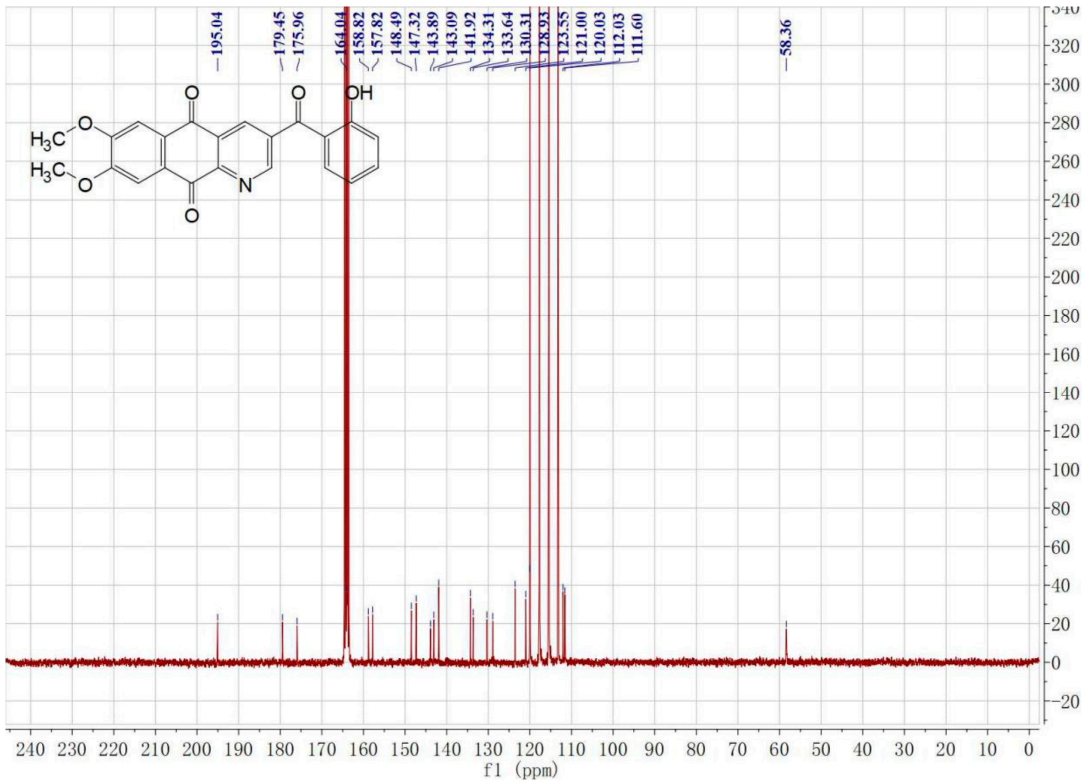

195.04
179.45
175.96
164.04
158.82
157.82
148.49
147.32
143.89
143.09
141.92
134.31
133.64
130.31
128.93
123.55
121.00
120.03
112.03
111.60
58.36
f1 (ppm)
